# *Pseudomonas aeruginosa* IscR-Regulated Ferredoxin NADP(+) Reductase Gene (fprB) Functions in Iron-Sulfur Cluster Biogenesis and Multiple Stress Response

**DOI:** 10.1371/journal.pone.0134374

**Published:** 2015-07-31

**Authors:** Adisak Romsang, Jintana Duang-nkern, Wilaiwan Wirathorn, Paiboon Vattanaviboon, Skorn Mongkolsuk

**Affiliations:** 1 Department of Biotechnology, Faculty of Science, Mahidol University, Bangkok, Thailand; 2 Laboratory of Biotechnology, Chulabhorn Research Institute, Bangkok, Thailand; 3 Center of Excellence on Emerging Bacterial Infections, Faculty of Science, Mahidol University, Bangkok, Thailand; 4 Program in Applied Biological Science: Environmental Health, Chulabhorn Graduate Institute, Bangkok, Thailand; 5 Center of Excellence on Environmental Health and Toxicology (EHT), Ministry Of Education, Bangkok, Thailand; CEA-Saclay, FRANCE

## Abstract

*P*. *aeruginosa* (PAO1) has two putative genes encoding ferredoxin NADP(+) reductases, denoted *fprA* and *fprB*. Here, the regulation of *fprB* expression and the protein’s physiological roles in [4Fe-4S] cluster biogenesis and stress protection are characterized. The *fprB* mutant has defects in [4Fe-4S] cluster biogenesis, as shown by reduced activities of [4Fe-4S] cluster-containing enzymes. Inactivation of the gene resulted in increased sensitivity to oxidative, thiol, osmotic and metal stresses compared with the PAO1 wild type. The increased sensitivity could be partially or completely suppressed by high expression of genes from the *isc* operon, which are involved in [Fe-S] cluster biogenesis, indicating that stress sensitivity in the *fprB* mutant is partially caused by a reduction in levels of [4Fe-4S] clusters. The pattern and regulation of *fprB* expression are in agreement with the gene physiological roles; *fprB* expression was highly induced by redox cycling drugs and diamide and was moderately induced by peroxides, an iron chelator and salt stress. The stress-induced expression of *fprB* was abolished by a deletion of the *iscR* gene. An IscR DNA-binding site close to *fprB* promoter elements was identified and confirmed by specific binding of purified IscR. Analysis of the regulation of *fprB* expression supports the role of IscR in directly regulating *fprB* transcription as a transcription activator. The combination of IscR-regulated expression of *fprB* and the *fprB* roles in response to multiple stressors emphasizes the importance of [Fe-S] cluster homeostasis in both gene regulation and stress protection.

## Introduction

Ferredoxin NADP(+) reductase (Fpr) is a flavin adenine dinucleotide (FAD)-containing oxidoreductase enzyme. It catalyzes reversible electron transfer between NADPH and electron carrier [Fe-S] proteins such as ferredoxin and flavodoxin [1, 2]. During photosynthesis, Fpr-ferredoxin complexes facilitate electron transfer from the Photosystem I to NADP^+^, generating NADPH for CO_2_ assimilation [3]. Bacterial Fprs also have ferric reductase activity [4]. Fpr also catalyzes a diaphorase reaction, which is an irreversible oxidation of NADPH in the presence of different electron acceptors such as ferricyanide, complexed transition metals, substituted phenols, nitro-derivatives, tetrazolium salts, viologens, quinones, and cytochromes [5].

Fprs are widely distributed in organisms ranging from bacteria to eukaryotes. These enzymes are divided into two main classes based on the structure, a plastid class and a bacterial class [6]. The plastid Fprs are found in photosynthetic organisms. Bacterial Fprs can be further subdivided into two subclasses. Subclass I Fprs have structures similar to that of the prototype *Azotobacter vinelandii* Fpr, while the structures of subclass II Fprs are homologous to that of the *Escherichia coli* Fpr [6, 7]. Among bacterial Fprs, subclass I Fpr typically contain less ferredoxin NADP(+) reductase catalytic efficiency than subclass II Fpr [4], and some Fprs have ferric reductase activity [4, 8]. The physiological role of Fpr in bacteria is unclear, though it has been shown to function in the bacterial oxidative stress response [4, 8]. Inactivation of subclass II *fpr* in *E*. *coli* increases sensitivity to paraquat (PQ), a superoxide-generating agent [8]. Increased expression of Fpr is associated with enhanced survival rate under superoxide stress [9]. The expression of *fpr* in *E*. *coli* and *Salmonella* is regulated by SoxRS proteins, transcriptional regulators that sense and respond to superoxide stress [9]. Moreover, *Pseudomonas putida* contains two Fprs, namely, *fprA* and *fprB*. The expression of *fprA* (encoding subclass I Fpr) is induced in response to superoxide stress and is regulated by FinR, a LysR-type transcriptional regulator [10]. Both FprA and FinR have important roles in cellular defense against stress due to superoxide and ferrous iron depletion [10–12]. The expression of *fprB* (encoding subclass II Fpr) is not affected by exposure to oxidants but is induced by osmotic stress [13]. The disruption of *fprB* in *P*. *putida* exhibits growth defects under high osmotic stress conditions and slower rate of recovery of oxidatively damaged aconitase (an [4Fe-4S]-containing enzyme) activity than that of the wild type [13].

Recently, Fpr has been shown to have roles in [Fe-S] cluster biogenesis in *E*. *coli*. One of the products from Fpr-catalyzed reactions is a reduced ferredoxin that is involved in [4Fe-4S] cluster biosynthesis [14]. Hence, the activities of [4Fe-4S] cluster-containing enzymes such as aconitase [15] and succinate dehydrogenase [16] and [4Fe-4S] transcription regulators such as an auto regulated *anr* that activates transcription of nitrate reductase genes such as *narG* [17–19] could be modulated by the availability of [4Fe-4S] clusters.

*Pseudomonas aeruginosa* is an opportunistic human pathogen that can thrive in harsh environments such as high temperature, high salt concentration and toxic chemicals. It is highly resistant to various chemical agents, such as antiseptics and several antibiotics. *P*. *aeruginosa* has several means of counteracting stressful conditions, including oxidative stress generated by innate immune cells or the environment. These factors are thought to contribute to bacterial virulence during the infection process [12]. The function of Fpr in *P*. *aeruginosa* is poorly understood. This bacterium contains two putative ferredoxin NADP(+) reductases: one Fpr is a member of bacterial subclass I and is denoted FprA (PA3397), and the other Fpr is a member of bacterial subclass II, which is named FprB (PA4615). Recently, FprB was proposed to promote Fenton chemistry in *P*. *aeruginosa* cells treated with oxidative stress-producing antibiotics via its ferric reductase activity [4, 12]. The *fprB* mutant displayed enhanced resistance to ampicillin, norfloxacin and gentamicin [12]. *P*. *aeruginosa* FprA can function as an electron donor to ferritin-like molecules, such as heme-binding bacterioferritins (Bfr) and non-heme-binding bacterial ferritins (Ftn), leading to release of the iron stored by these ferritins into the cytoplasm [20]. Under iron starvation, however, FprA is an electron donor to heme oxygenase, an enzyme involved in heme metabolism [21].

In this study, the physiological function of *fprB* in *P*. *aeruginosa* was evaluated through phenotypic analysis of the mutant. Our data indicated that *fprB* has defects in [Fe-S] cluster biogenesis and that Fpr activity is required for survival under oxidative, osmotic, and metal stresses. Unlike other bacteria, the expression of *fprB* is controlled by IscR, a global transcriptional regulator of genes involved in [Fe-S] cluster biogenesis and stress responses.

## Results and Discussion

### PAO1 *fprB* gene and transcription organization

**Fig 1:**
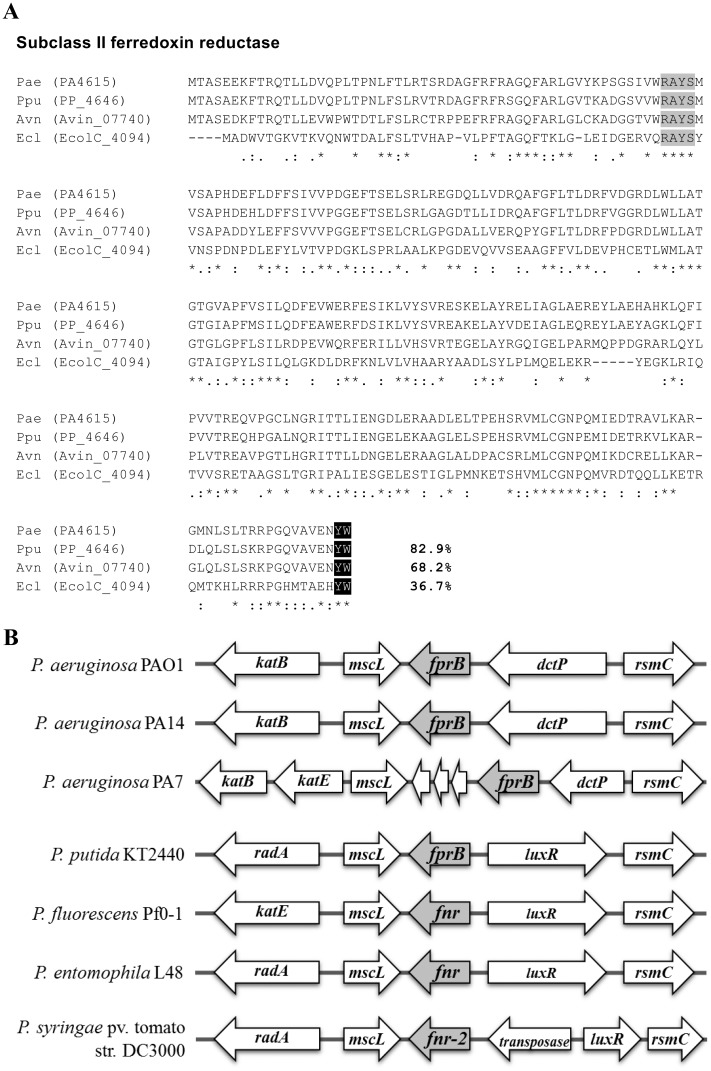
Multiple amino acid sequence alignment and gene organization of *P*. *aeruginosa fprB*. (A) Alignment of *P*. *aeruginosa* FprB with other characterized bacterial FprB enzymes (Ppu, *P*. *putida*; Avn, *A*. *vinelandii*; and Ecl, *E*. *coli* ATCC 8739) was performed using the CLUSTALW program [57]. Black (YW) and light gray (RAYS) boxes indicate the subclass II signature amino acid and the FAD-binding domain, respectively. The asterisk, colon, and period symbols indicate identical residues, conserved substitutions, and semi-conserved substitutions, respectively. Number indicates percent identity of the aligned protein with that of *P*. *aeruginosa*. (B) Gene organization at the *fprB* locus among *Pseudomonas* spp. *fnr* and *fnr-2*, *fprB*-homologous genes; *katB* and *katE*, catalase genes; *mscL*, gene encoding large conductance mechanosensitive channel; *dctP*, gene encoding propable periplasmic C4-dicarboxylate binding-protein; *rsmC*, gene encoding propable 16S RNA G1207 methylase; *luxR*, LuxR family DNA-binding transcriptional regulator gene; *radA*, gene encoding propable DNA repair protein; non-labelled, gene with unknown annotation.

Analysis of the *P*. *aeruginosa* PAO1 genome database [22] identified two genes, *fprA* (PA3397) and *fprB* (PA4615), encoding putative ferredoxin NADP(+) reductase similar to that of *A*. *vinelandii* and *P*. *putida* genomes. *P*. *aeruginosa* FprA shares low (33.7%) amino acid identity with FprB (S1 Fig). *P*. *aeruginosa* FprB, a 285-amino acid protein, shares high amino acid identity with FprB from *P*. *putida* (82.9%) [13] and *A*. *vinelandii* (68.2%) [23] (Fig 1A), while it shares 36.7% identity with *E*. *coli* Fpr [8]. The major differences between Fpr sub-classes I and II are found in the C-terminal region [6]. The signature amino acid residues for Fpr sub-class II, which include the tyrosine-247 [24] and tryptophan-248 residues of *E*. *coli* Fpr [6], are conserved in *P*. *aeruginosa* FprB (Fig 1A). Thus, similar to *P*. *putida* FprB, the *P*. *aeruginosa* FprB belongs to subclass II of bacterial ferredoxin NADP(+) reductases.

The locations of *fprB* in genomes of different *Pseudomonas* species are varied. In most *Pseudomonas spp*., including *P*. *putida*, *P*. *fluorescens*, *and P*. *entomophila*, *fprB* is located between *mscL*, encoding a large conductance mechano-sensitive channel, and an open reading frame (ORF) encoding a homolog of the LuxR transcription regulator (Fig 1B). Nevertheless, the role of this LuxR family of transcription regulators in regulation of *fprB* in *P*. *putida* is unclear [13]. In *P*. *syringae* genome, a gene encoding putative transposase was inserted between *fprB*-homolog (*fnr-2*) and *luxR* (Fig 1B). Whereas, in *P*. *aeruginosa* strains such as PAO1 and PA14, *fprB* is located between *mscL* (PA4614) and PA4616, encoding a probable C4-dicarboxylate-binding protein (Fig 1B). Nonetheless, analysis of different isolates of *P*. *aeruginosa* shows significant variation in the genomic location of *fprB*. For example, *P*. *aeruginosa* PA7 genome contains three unknown open reading frames inserted between *fprB* and *mscL* genes (Fig 1B). Our Northern blot analysis results (in the expression analysis section) indicated that PAO1 *fprB* is transcribed as a monocistronic mRNA.

### Expression analysis of *fprB*

**Fig 2:**
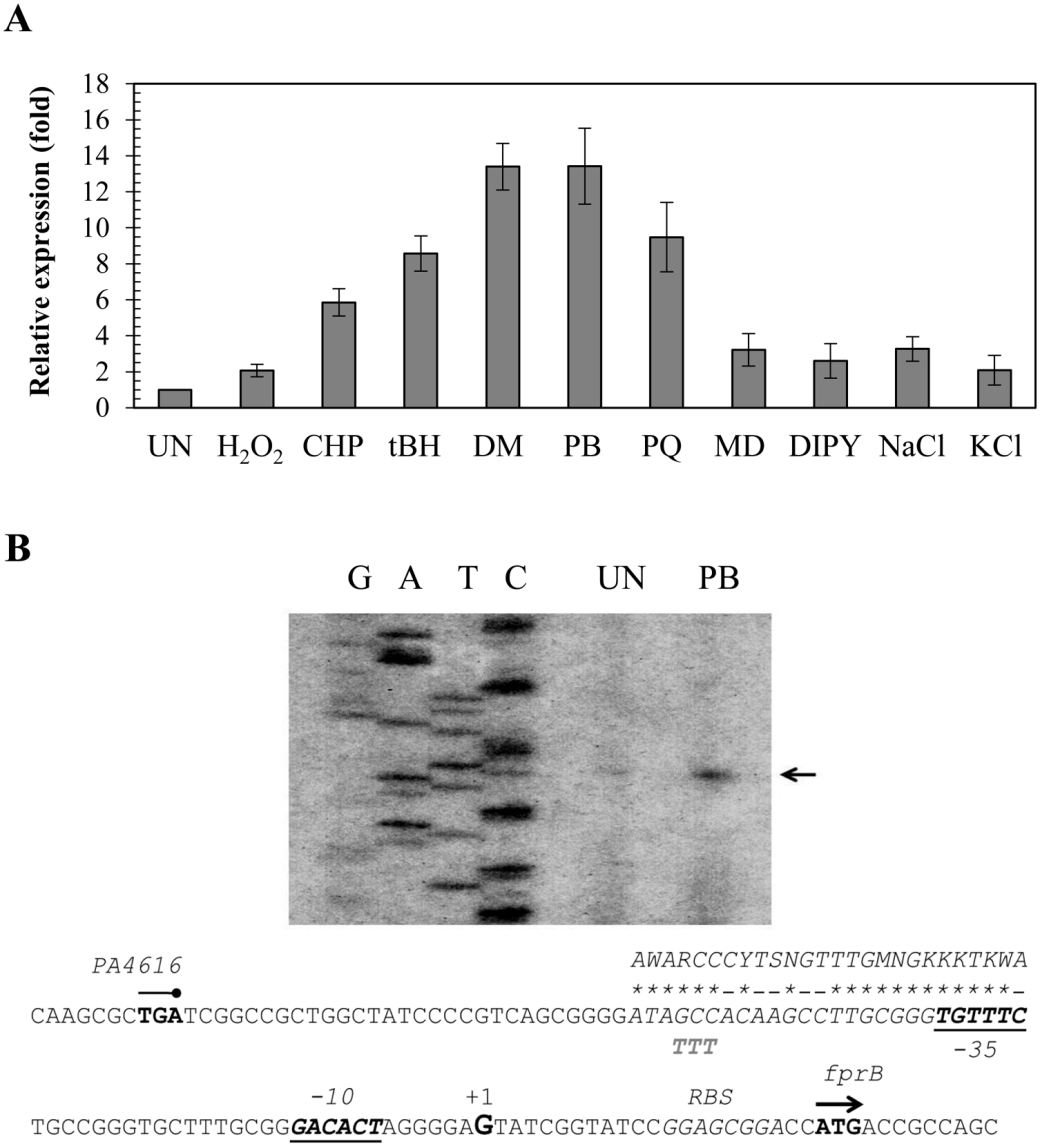
Expression profile and characterization of *fprB* promoter. (A) Real-time RT-PCR analysis of *fprB* expression. The PAO1 cultures were induced with 1 mM H_2_O_2_, 1 mM CHP, 1 mM tBH, 1 mM DM, 0.5 mM PB, 0.5 mM PQ, 0.5 mM MD, 1 mM DIPY, 5 mM NaCl or 5 mM KCl for 15 min prior to RNA preparation and real-time RT-PCR analysis as described in the experimental procedures. The data shown are the means and SD of three independent experiments. (B) Promoter characterization using primer extension assay. Reverse transcription was performed using ^32^P-labeled BT3552 primer and RNA extracted from PAO1 cultured under uninduced (UN) conditions or 0.5 mM PB. The extension products were separated on an 8% acrylamide-7 M urea sequencing gel. G, A, T, C represent the DNA sequence ladder prepared using a sequencing kit (Epicentre) with ^32^P-labeled primer and the putative *fprB* promoter fragment as template. The arrowhead points to the transcription start site (+1). The putative -10 and -35 elements are underlined. The consensus sequence of the *E*. *coli* IscR binding site is aligned above the sequence line in corresponding letters, and the homologous bases are marked by asterisks. The mutagenized bases (from GCC to TTT) in the putative IscR binding motif are shown below the sequence line.

*P*. *aeruginosa* FprB might has important roles in maintaining functional levels of [4Fe-4S] clusters. The regulation of its expression is therefore also likely to be important, especially during stressful growth conditions. The *fprB* expression profile was determined using end-point and real-time RT-PCR. Initially, the expression of *fprB* was monitored throughout all phases of bacterial growth, and no significant changes in *fprB* expression levels were observed during different growth phases (data not shown). Oxidative stress is known to affect the balance of [Fe-S] clusters, and hence, *fprB* expression levels in response to sources of oxidative stress were determined. Exposure of PAO1 to redox cyclers such as PB (0.5 mM), PQ (0.5 mM) and MD (0.5 mM) induced *fprB* expression at levels that ranged from 4- to 14-fold increases over the uninduced levels (Fig 2A). The effects of exposure to various peroxides were also investigated. Exposure to H_2_O_2_ (1 mM) produced less than a 2-fold induction, while treatment with organic hydroperoxides (tBH, 1 mM and CHP, 1 mM) induced 6- to 8.5-fold increases, respectively. DM (1 mM) induced a 14-fold increase in *fprB* expression (Fig 2A). Moreover, growth under low intracellular iron conditions (via addition of DIPY, 1 mM) also produced a low level (3-fold) of induction of gene expression (Fig 2A). Osmotic stress (excess NaCl or KCl) induced 2-3-fold increases in *fprB* expression (Fig 2A). The *fprB* expression profile clearly shows that treatment with redox cycling compounds and an electrophile highly induced expression levels (over 10-fold), while organic hydroperoxides (H_2_O_2_), low iron and osmotic stress resulted in moderate (6-8-fold) to low levels (2-3-fold) induction of expression. The stress-induced expression profile of *P*. *aeruginosa fprB* was unlike previously observed patterns in *P*. *putida*, where *fprB* expression was inducible by osmotic stress conditions but not by oxidative stress conditions [13]. The expression pattern of *P*. *aeruginosa fprB* supports its role in oxidative, osmotic and metal stress responses. Under these conditions, there is a higher demand for [Fe-S] cluster biogenesis and cluster repair, hence the corresponding increase in *fprB* expression. The induction of *fprB* expression by iron depletion also supports the requirement of FprB for [Fe-S] cluster biogenesis [21, 25].

### *fprB* is regulated by IscR

The pattern of *fprB* expression in response to redox cycling agents and peroxides suggests that it could be regulated by a global superoxide–peroxide stress sensor and transcription regulator. The analysis of *fprB* promoter and transcription-regulator binding motifs could reveal information about the mode of regulation of the gene. Primer extension experiments were carried out to determine the 5’ end of *fprB* transcripts from RNA samples isolated from uninduced and PB-treated cultures. The primer extension product in both RNA samples was mapped to a G residue located 21-bp upstream of the *fprB* start codon (ATG) (Fig 2B). The *fprB* promoter motifs at the -35 and -10 regions were deduced from the transcription start site as TGTTTC and GACACT, respectively, separated by 17 nucleotides. Primer extension results also confirmed the real-time RT-PCR results that PB (0.5 mM) is a potent inducer of *fprB* expression, as shown by over 10-fold increase in the amount of primer extension product (Fig 2B). The data also suggest that the observed induction by PB is likely due to an increase in transcription of the gene from its promoter.

*E*. *coli fpr* (an *fprB* homolog) is a member of the SoxRS regulon [1]. The *P*. *aeruginosa* SoxR paradigm is different from the *E*. *coli* SoxR paradigm, in that *P*. *aeruginosa* SoxR directly regulates its target genes [26]. SoxR-regulated genes share conserved SoxR boxes located between promoter elements in a diverse group of bacteria [27, 28]. No putative SoxR binding site (CCTCAAGtttgCTTGAGG) [29] could be identified near the *fprB* promoter region, suggesting that SoxR does not participate in the regulation of *fprB*.

**Fig 3:**
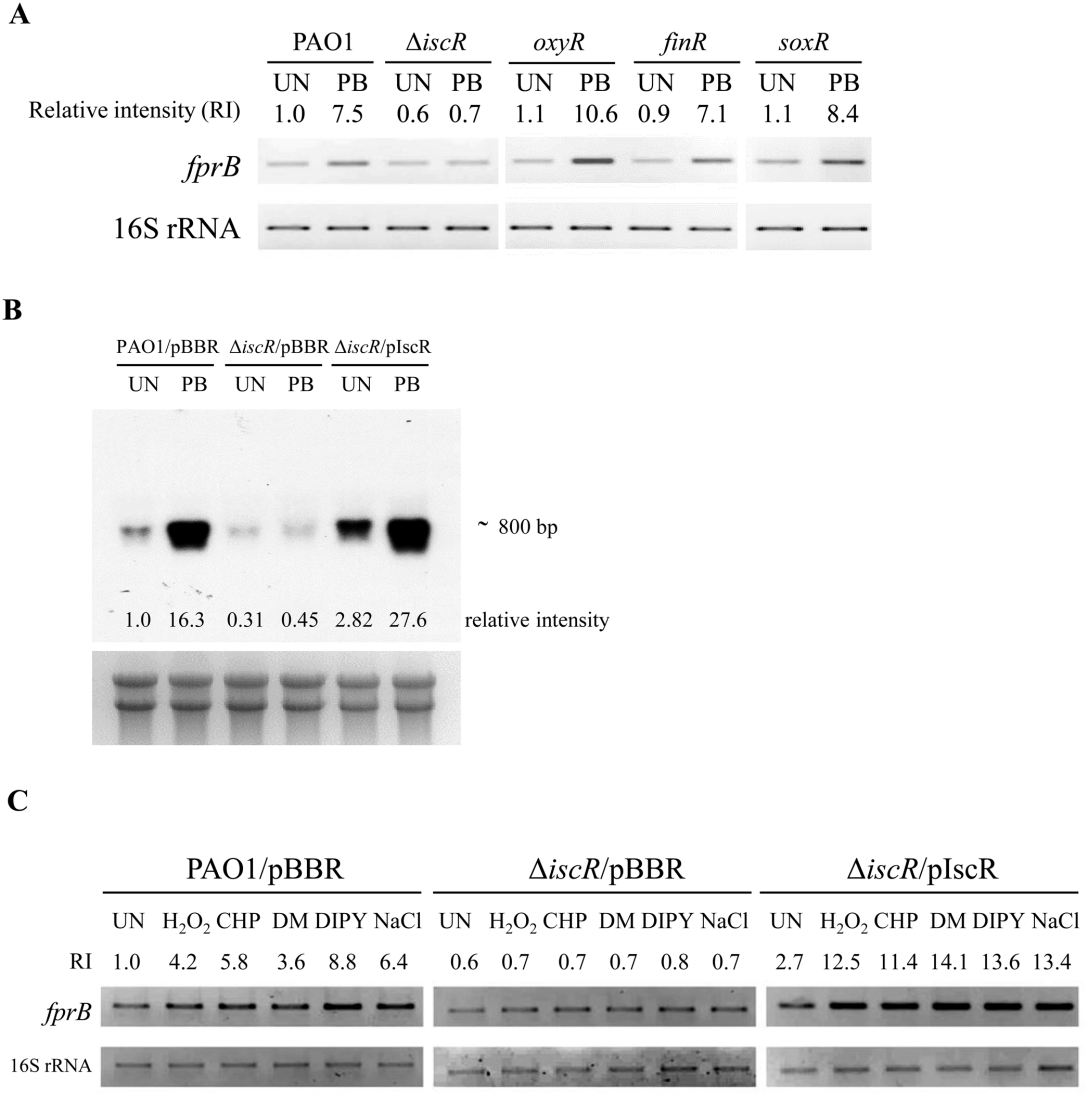
End-point RT-PCR and Northern blot analyses of *fprB* expression in *P*. *aeruginosa* strains. (A) The *fprB* expression profiles in PAO1, Δ*iscR*, *oxyR*, *finR*, and *soxR* mutants were determined using end-point RT-PCR. Exponential cells were grown at 37°C under uninduced (UN) or induced conditions with 0.5 mM plumbagin (PB) for 15 min. (B) The *fprB* expression profiles in PAO1 harboring empty vector (PAO1/pBBR), Δ*iscR* mutant harboring empty vector (Δ*iscR* /pBBR), and the complemented Δ*iscR* mutant (Δ*iscR* mutant/pIscR) were analyzed using Northern blot. Bacteria were cultured under uninduced (UN) or induced conditions with 0.5 mM PB. (C) The *fprB* expression profiles in PAO1/pBBR, Δ*iscR* /pBBR, Δ*iscR* mutant/pIscR in response to reagents (i.e., 0.25 mM PB, 1 mM H_2_O_2_, 1 mM CHP, 1 mM DM, 1 mM DIPY, and 0.25 M NaCl) were determined using end-point RT-PCR. All data presented in this figure were representatives of triple biologically independent replications and the number indicated above or below each band represents the fold change in band intensity relative to a level of the uninduced culture of PAO1/pBBR, determined using densitometric analysis.

In *P*. *aeruginosa*, FinR, IscR, OxyR and SoxR have been shown to be sensors of superoxide-peroxide stresses [29–32]. In an attempt to identify the transcriptional regulator responsible for the regulation of *P*. *aeruginosa fprB*, expression analysis was repeated in PAO1, *finR*, *iscR*, *oxyR and soxR* mutants cultured in medium with and without PB. The expression of genes under the regulation of these genes is known to be highly induced upon challenge with redox cycling drugs [29–32]. End-point RT-PCR analysis revealed that PB-induced expression of *fprB* was presented at similar levels to PAO1 in *finR*, *oxyR*, *soxR* mutants, indicating that these regulators were not responsible for the PB-inducible expression of *fprB* (Fig 3A). In contrast, PB-induction of *fprB* expression was abolished in the Δ*iscR* mutant and was restored in the Δ*iscR* mutant complemented with a pIscR plasmid (Fig 3B and 3C). Similarly, the induction of *fprB* expression by other oxidants (H_2_O_2_, CHP, DM) and DIPY treatments were also abolished in the Δ*iscR* mutant (Fig 3C). The induction of *fprB* by these oxidants in the Δ*iscR* mutant could be restored in the Δ*iscR*/pIscR strain (Fig 3C). Northern blot analysis of *fprB* expression was performed on RNA samples purified from PAO1/pBBR, the Δ*iscR/*pBBR mutant and the complemented Δ*iscR* mutant (Δ*iscR*/pIscR) [32, 33] cultured with and without PB. The Northern blot results clearly show that PB highly induces *fprB* expression in PAO1 and that this PB induction of gene expression was abolished in the Δ*iscR* mutant, confirming the RT-PCR results (Fig 3B). The PB induction of *fprB* in the Δ*iscR* mutant could be restored in the complemented Δ*iscR*/pIscR strain (Fig 3B). The results indicate that IscR is the transcription regulator responsible for oxidant-inducible expression of *fprB*.

Further analysis of *fprB* expression data revealed that IscR is a transcription activator of *fprB*. Northern blot analysis of *fprB* expression showed that the basal, uninduced level of *fprB* expression in the Δ*iscR* mutant was 3.2-fold lower than the level in PAO1 (Fig 3B) and that in a complemented Δ*iscR*/pIscR strain, *fprB* was expressed at a higher (2.82-fold) level than the level in PAO1 (Fig 3B). The PB-induced level in the complemented strains (Δ*iscR*/pIscR) was also increased slightly (69%) over the induced levels in PAO1 (Fig 3B). Similar patterns of *fprB* expression in Δ*iscR* mutant and the complemented strains were observed using end-point RT-PCR analysis (Fig 3A and 3C). These data suggest that IscR acts as a transcriptional activator of *fprB* and regulates gene expression in response to redox cycling drugs. Moreover, *fprB* is the first gene in *Pseudomonas* that is shown to be activated by IscR.

**Fig 4:**
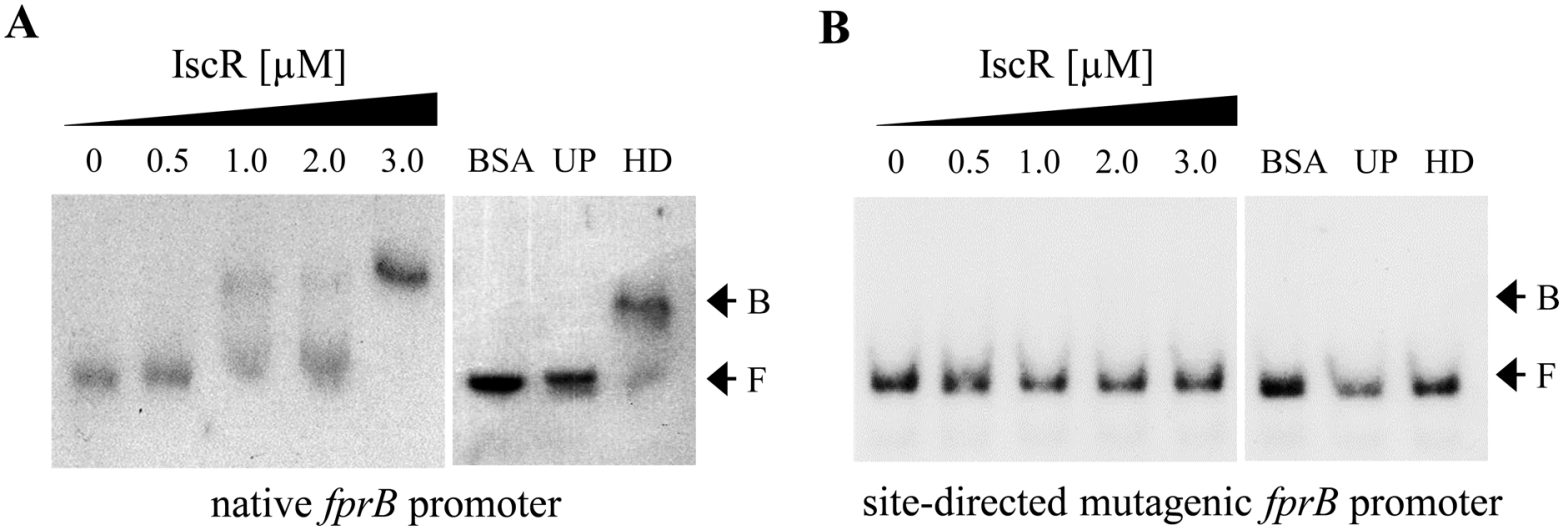
Gel mobility shift assay. Purified protein preparation and protein-DNA binding assay were performed as described in the experimental procedures. The gel mobility shift assay was performed using the ^32^P-labeled native (A) and mutagenic (B) *fprB* promoter fragment and increasing concentrations of purified IscR. UP and HD represent the addition of 1 μg unlabeled *iscR* promoter and 2.5 μg of heterologous DNA (pUC18 plasmid), respectively, to the binding reaction mixtures containing 3.0 μM IscR. F and B indicate free and bound probes, respectively. The data presented was a representative of three biologically independent experiments.

The are two types of IscR binding site namely type I and type II, these sites are bound differentially by holo-IscR and apo-IscR and either repress or activate target gene expression [34, 35]. A search for an IscR box near the *fprB* promoter revealed a putative IscR-binding site with the sequence 5’ATAGCCACAAGCCTTGCGGGTGTTTC3’ located adjacent to the putative -35 region of the promoter (Fig 2B). This sequence box shares 78% identity with the type-II *E*. *coli* IscR binding motif (AWARCCCYTSNGTTTGMNGKKKTKWA) while it shares 44% and 52% identities with the PAO1 IscR binding motif (ATAGTTGACCNATTTTCTCGGGNAA) [32] and the type-I *E*. *coli* IscR binding motif (ATASYYGACTRWWWYAGTCRRSTAT) [34], respectively. This result supports the direct regulation of *fprB* by IscR and enhances a possibility that IscR binds this putative box near the *fprB* promoter as the type-II binding manner. The regulation of *fprB* by IscR was further investigated. A gel mobility shift assay was conducted using purified *P*. *aeruginosa* IscR protein with a C-terminal 6×His tag [33] and a 184-bp DNA fragment spanning the *fprB* regulatory region. The results demonstrated that IscR binds specifically to the identified *fprB* regulatory DNA fragment (Fig 4A). Excess unlabeled probe (UP), but not heterologous DNA (HD), could compete with the labeled probe in the IscR-binding complex formation. No binding complexes were detected when excess unrelated protein was added to the labeled probe instead of purified IscR in a binding reaction (Fig 4A). To assess the role of the putative IscR-binding site of the *fprB* promoter, PCR-based site-directed mutagenesis of the putative IscR-binding motif was performed using the *fprB* promoter fragment as a template. The highly conserved bases GCC of IscR binding motif of *E*. *coli* [34] in the putative IscR binding sequence 5’ATAGCCACAAGCCTTGCGGGTGTTTC3’ were mutated to TTT. The gel mobility shift experiments were performed using mutated *fprB* promoter, where the GCC sequence in the putative type-II IscR binding site was replaced with TTT. As shown in Fig 4B, no shift complexes were observed, indicating that mutagenesis of the putative IscR binding sequence abolished the binding ability of purified IscR. These results support the hypothesis that IscR directly binds to the *fprB* promoter at the proposed IscR binding site and regulates *fprB* expression. This is the first report of a homologous sequence of type-II IscR-binding site in *P*. *aeruginosa* genome. In *E*. *coli*, [2Fe-2S] IscR binds to both types of binding motifs, whereas apo-IscR binds to type-II motifs [36]. Spectral analysis of purified IscR used in this study had roughly 50% presence of [Fe-S] cluster [22]. Hence it was not possible to differentiate the specific protein type that binds to this proposed box near the *P*. *aeruginosa fprB* promoter. However, the type-II IscR-binding mechanism in *P*. *aeruginosa* is under investigation.

The regulation of *fprB* by IscR, an important regulator of Fe-S biogenesis and stress response, illustrates the importance of co-ordination between the gene regulation and physiological roles in the stress response. IscR negatively auto-regulates the *isc* operon and positively regulates *fprB*. Thus, the levels of IscR are important in determining the expression levels of the genes that balance [Fe-S] cluster availability and the overall ability of cells to cope with multiple stresses. *E*. *coli* apo-IscR binds type-II sites on the *suf* operon promoter and promotes the Fe-S biogenesis under stress conditions [34]. However, no homologues of the complete *suf* operon could be identified in the PAO1 genome [22] suggesting that FprB involves in the Fe-S biogenesis through the ISC system. Under low iron and oxidative stress conditions, levels of [Fe-S] cluster decreased leading increased levels of apo-IscR in the cell. This results in depression of *isc*-operon and increased concentrations of IscR leading to activation of *fprB* in order to increase the [Fe-S] cluster biogenesis.

### Inactivation of *fprB* diminishes the activities of [Fe-S] cluster-containing enzymes

**Fig 5:**
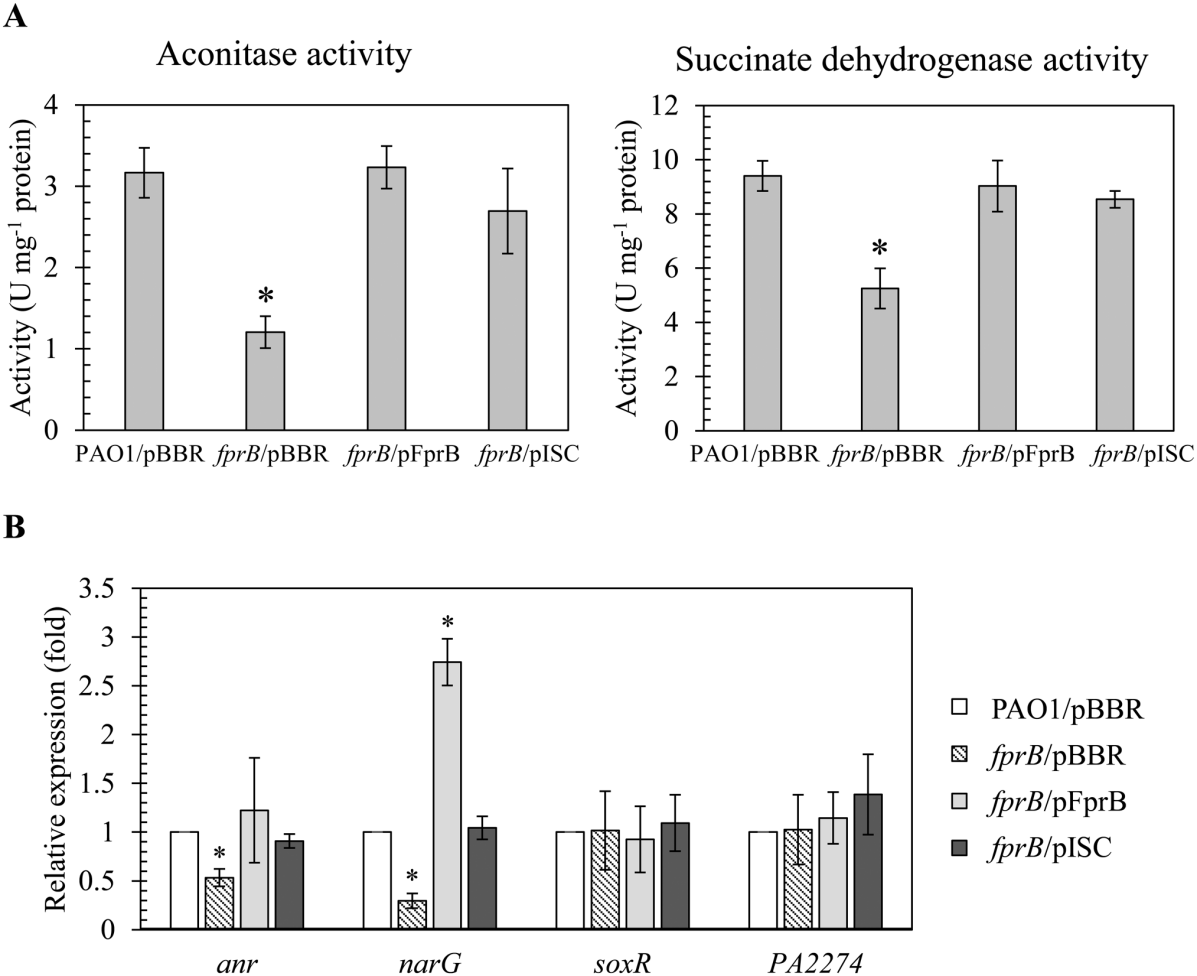
Iron-sulfur cluster-containing protein activities. (A) Aconitase and succinate dehydrogenase (Sdh) activities in *P*. *aeruginosa* PAO1 and *fprB* mutant harboring empty vector (pBBR), pFprB, or pISC strains under normal growth condition were measured. Cell culture conditions, protein preparation and enzymatic activity assays were performed as described in the experimental procedures. The data shown are means and SD of triple biologically independent replications. The asterisk indicates a statistically significant difference (P < 0.01) compared with the PAO1/pBBR strain and pBBR referred as an empty vector control. (B) Real-time RT-PCR analysis of *anr*, *narG*, *soxR* and PA2274 in indicated *P*. *aeruginosa* strains cultivated under normal growth condition. RNA isolation and real-time RT-PCR were performed as described in the experimental procedures. The data shown are means and SD of three independent experiments. The asterisk indicates a statistically significant difference (P < 0.01) compared to those of the PAO1/pBBR strain.

Here, we assessed the effect of *fprB* inactivation on the overall [Fe-S] cluster balance of the cell. The status of [Fe-S] clusters was assessed in the mutant and a parental strain. First the status of [4Fe-4S] was assessed by determining the activity levels of the [Fe-S] cluster-containing enzymes aconitase and succinate dehydrogenase (Sdh) in the *fprB* mutant and PAO1 [15, 16]. In addition, the relative expression of [4Fe-4S] cluster containing transcription regulator *anr* and one of its target gene *narG* [17–19] was monitored in the *fprB* mutant and a parental strain. The results of enzyme assays indicated that the activities of both aconitase and Sdh were reduced by approximately 50% in the *fprB* mutant relative to the PAO1 wild type under normal growth condition (Fig 5A). This reduction in the mutant enzyme activities could be fully complemented to wild-type levels by pFprB, an expression vector containing functional *fprB* (Fig 5A). In addition, the relative expression of *anr* and *narG* were reduced by 50 to 70% in the *fprB* mutant compared to the PAO1 strain (Fig 5B). The reduction in transcription activities of these genes in the mutant could be complemented by pFprB to PAO1 levels (Fig 5B). Moreover, expression of *fprB* from the expression vector resulted in two to three fold increased in the relative expression of *narG* over PAO1 levels (Fig 5B).

To test whether decreases in these enzymes’ activities were resulted from the lack of active cofactor [4Fe-4S] cluster or not, pISC, an expression vector containing functional *iscASU-hscBA-fdx2-iscX* (genes in the *P*. *aeruginosa isc*-operon encoding necessary machineries for [Fe-S] cluster biogenesis), was transformed into the mutant and the enzymatic activities and transcription activities of the genes were monitored in these strains. Fig 5A showed that the *fprB* mutant harboring pISC exhibited an approximately 85% of aconitase and Sdh activities relative to the PAO1 wild type levels and has similar level of these enzymatic activities as the *fprB* mutant harboring pFprB. The reduction in transcription levels of *anr* and *narG* in the mutant was complemented to PAO1 levels in *fprB/*pISC strain (Fig 5B).

Subsequently, the status of [2Fe-2S] clusters in the mutant was determined by measuring the expression of *soxR* mRNA (encoding a [2Fe-2S] cluster-containing transcriptional regulator) and its target gene PA2274 by real-time RT-PCR. The data show that *fprB* inactivation has no effect on expression levels of an auto-regulated *soxR* or the SoxR-targeted gene, PA2274 relative to wild type (Fig 5B). The similar functionality of [2Fe-2S] cluster-containing proteins in the mutant and PAO1 suggests that FprB may not be involved in the maintenance of [2Fe-2S] cluster status and its inactivation does not significantly alter the physiological levels of [2Fe-2S] clusters.

The results suggest that FprB could be involved in [4Fe-4S] cluster biogenesis pathway and required for full activity of [4Fe-4S] containing enzymes and transcription regulators. FprB is catalyzed the reaction leading to formation of reduced ferredoxin, a key electron donor in the biogenesis of [4Fe-4S] cluster from [2Fe-2S] cluster [14]. Inactivation of FprB leads to decrease [4Fe-4S] cluster biogenesis, resulting in an overall decrease in the functional levels of [4Fe-4S] cluster, reflected in the observed activity reductions in aconitase and Sdh. The reduced transcription activities of [4Fe-4S] cluster-containing transcription regulator *anr* and its target gene *narG* further supported the role of *fprB* in [4Fe-4S] cluster biogenesis. Recently, *E*. *coli* ferredoxin, encoded by *fdx2* in an *isc*-operon, functions in conjunction with Fpr and NADPH to transfer electrons to promote [Fe–S] cluster biogenesis [14]. The results support the notion that FprB has important role in the [4Fe–4S] cluster biogenesis. In addition, it has been proposed that *P*. *putida* FprB has the ability to repair damaged [4Fe-4S] clusters [13]. Thus, in the mutant, reduced cluster repair could further reduce the physiological, functioning levels of [4Fe-4S] cluster and potentiated the sensitivity to stresses. The mechanisms of *P*. *aeruginosa* FprB for repairing [Fe-S] clusters are under investigating.

### The physiological role of *fprB* in the oxidative stress response

**Fig 6:**
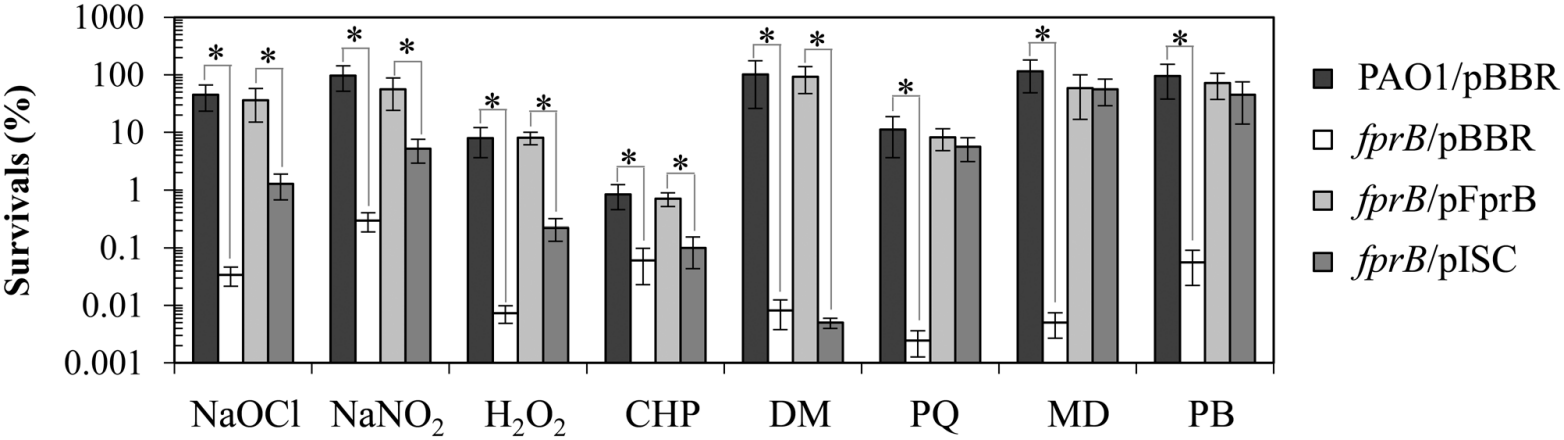
Determination of oxidant resistance levels of *P*. *aeruginosa* strains. The oxidant resistance levels in *P*. *aeruginosa* PAO1 and *fprB* mutants that harbored either empty vector (pBBR), pFprB (for *fprB* expression), or pISC (for expression of the functional *isc* operon without *iscR*). The resistance levels against NaOCl (0.045%) and NaNO_2_ (pH 5.0, 50 μM) were determined using a bacterial killing assay while those against other oxidants i.e., H_2_O_2_ (0.5 mM), cumene hydroperoxide (CHP, 1.6 M), diamide (DM, 13 mM), paraquat (PQ, 200 μM), menadione (MD, 4 mM), and plumbagin (PB, 1 mM) were performed using a plate sensitivity assay as described in the experimental procedures. The data shown are means and SD of percent survival at 18 h incubation from three independent experiments. The asterisk indicates a statistically significant difference (P < 0.01) between the two indicated strains.

The physiological role of FprB in protection against oxidative stress was investigated using an *fprB* mutant and a plate sensitivity assay and exposure to various oxidative stress conditions. The *fprB* mutant was 10^2^- and 10-fold more sensitive to treatment with hydrogen peroxide (H_2_O_2_, 0.5 mM) and organic hydroperoxides (cumene hydroperoxide [CHP], 1.6 mM), respectively (Fig 6). The mutant was highly sensitive to treatment with redox cycling/superoxide generating-compounds PQ (200 μM), menadione (MD, 4 mM), and plumbagin (PB, 1 mM), which caused over 10^4^-fold reduction in survival percentage compared to PAO1 (Fig 6). Additionally, the mutant was 10^4^-fold more susceptible to a thiol-depleting agent diamide (DM, 13 mM) than PAO1 (Fig 6). The resistance of the mutant to other oxidative and nitrosative stresses was then measured, including a strong oxidant, sodium hypochlorite (NaOCl), and a generator of nitrosative stress, acidified sodium nitrite (NaNO_2_). The results showed that the *fprB* mutant was over 10^3^- and 10^2^-fold more sensitive to treatments with either 0.045% NaOCl or 50 mM NaNO_2_ pH 5.0, respectively, than PAO1 (Fig 6). The observed phenotypes of the *fprB* mutant are different from those of the closely related *P*. *putida fprB* mutant, which shows no alteration in sensitivity to PQ-induced oxidative stress [13]. However, *fpr* inactivation in *E*. *coli* does result in increased sensitivity to oxidative and nitrosative stress [1]. Overall, the results indicate that in *P*. *aeruginosa*, FprB has a crucial role in bacterial survival under oxidative stress and nitrosative stress conditions.

Inactivation of *fprB* showed varying degrees of sensitivity increases to different stresses tested. The nature of these stresses was diverse, and it is likely that the sensitivity arose from a general defect of cell function. Our observations showed that the *fprB* mutant had reduced activities of [4Fe-4S] cluster-containing enzymes and transcription factor (Fig 5). The reduced activities of these [4Fe-4S] clusters-containing proteins could be restored fully by expression of *fprB* and partially by pISC. Hence, the defect in [Fe-S] cluster biogenesis is possibly responsible for the increased sensitivity to multiple stresses in the *fprB* mutant. Experiments were performed to test whether the mutant phenotypes of increased sensitivity to multiple oxidative stresses result from a reduced availability of [Fe-S] supply for cellular activities. We tested whether increasing levels of [Fe-S] cluster biogenesis genes could compensate for the lack of FprB in protecting the bacteria against oxidants was performed by transforming pISC into the *fprB* mutant. This allows direct determination of the roles [Fe-S] biogenesis in alleviating oxidant sensitivity of the *fprB* mutant. In *E*. *coli*, a small regulatory non-coding RNA RyhB binds to the second cistron of the *iscRSUA* mRNA and promotes the cleavage of the downstream *iscSUA* transcript but no putative *ryhB* or *P*. *aeruginosa* sRNA *prrF* (a functional *ryhB* analogue) binding motifs were identified between the *iscR* and *iscS* intergenic sequences of *P*. *aeruginosa* PAO1. The sensitivity levels were measured in the *fprB* mutant and PAO1 strains harboring either pISC, pFprB or an empty vector control. As expected, expression of *fprB* from a vector (*fprB*/pFprB) fully complemented all oxidant-sensitive phenotypes of the mutant and restored sensitivity levels to those attained in PAO1 (Fig 6). This experiment confirmed that the oxidant-sensitive phenotype arose directly from a lack of FprB. Similarly, the oxidants could be divided into three groups based on their effects on the sensitivity levels of *fprB*-harboring pISC (*fprB*/pISC) strain. The first group of oxidants, including an organic hydroperoxide (CHP) and an electrophile (DM), produced similar levels of sensitivity in *fprB*/pISC and control strains (Fig 6). Treatment of *fprB*/pISC with the second group of oxidants, including NaOCl, NaNO_2_, and H_2_O_2_, produced an intermediate level of protection, lower than the resistance of PAO1 but higher than that of the *fprB* mutant (Fig 6). The presence of pISC fully protected *fprB* mutant from MD, PQ and PB treatments, allowing resistance of mutant to reach the level attained by PAO1 wild type (Fig 6).

It has been suggested that *P*. *putida* FprB promotes Fenton chemistry by iron mobilization from storage proteins and by ferric reductase activity, which generates ferrous ions from ferric ions in the presence of H_2_O_2_, leading to hydroxyl radical production and ultimately contributing to cell death [12]. If iron mobilization and ferric reductase activities by FprB in *P*. *aeruginosa* have major roles in the oxidative stress response, then inactivation of *fprB* should lead to increased resistance to H_2_O_2_. On the contrary, the *P*. *aeruginosa fprB* mutant was more susceptible to H_2_O_2_ treatment, suggesting that iron mobilization and ferric reductase activity are not responsible for the *fprB* mutant phenotype. Alternatively, Fpr may use NADPH to generate active peroxide scavengers that function in the detoxification of H_2_O_2_ and CHP [37]. Accordingly, *fprB* inactivation led to increased sensitivity to peroxides. Increased sensitivity to nitrosative stress (acidified nitrite) of the *P*. *aeruginosa fprB* mutant is not unexpected. Under acid condition, nitrite is protonated to produce HNO_2_ that would undergo dismutation, generating nitric oxide radical (NO) [38].

[Fe-S] clusters are targets for ROS [39, 40]. Both H_2_O_2_ and superoxide can oxidize solvent-exposed labile [4Fe-4S]^2+^ clusters such as those of dehydratases, leading to the formation of [3Fe-4S]^1+^ and the release of Fe, resulting in inactivation of enzymatic activity. In addition, H_2_O_2_ can attack nascent [Fe-S] clusters on carrier proteins during [Fe-S] cluster assembly [41]. Analysis of *fprB* and [Fe-S] cluster biogenesis in protection against oxidative stress revealed multiple and diverse roles of the enzyme in the process. First, the increased sensitivity of the mutant to the organic hydroperoxide CHP did not result from defects in [Fe-S] cluster biogenesis, because increased expression of *isc*-operon genes did not affect mutant sensitivity (Fig 6). Similarly, the sensitivities to diamide could not be complemented by pISC. This result suggests that [Fe-S] cluster biogenesis defects are not responsible for the *fprB* mutant sensitivity phenotype, suggesting that additional mechanisms partly contribute to the phenotypes of the mutant, such as oxidation of protein thiols by diamide at active sites or sites of structural importance and subsequent loss of function. Increasing [Fe-S] cluster biogenesis did partly complement the sensitivity phenotype of the mutant in response to H_2_O_2_, NaOCl and NaNO_2_, implying that a decreased [Fe-S] cluster pool contributes to sensitivity of the mutant to these oxidants. Factors that may contribute to non- or partial complementation of the *fprB* oxidant-sensitive phenotype by pISC could be because [Fe-S] cluster assembly requires genes such as *grxD* (monothiol glutaredoxin) and *nfuA* ([Fe-S] cluster carrier protein), which are located outside the *isc* operon [42, 43]. In addition to the [Fe-S] cluster-related mechanism responsible for the phenotype, ROS can react with mononuclear iron proteins, leading to inactivation via the Fenton reaction. Fpr allows repair of oxidative damage to [Fe-S] clusters [19]. [4Fe-4S] cluster-containing hydratase enzymes such as aconitase or 6-phosphogluconate dehydratase could be reactivated by reactions catalyzed by Fpr [9]. The recovery of oxidatively damaged aconitase activity was slower for the *fprB* mutant than that for the wild type in *P*. *putida* KT2440 [13]. Nonetheless, the mutant could carry out the reactivation at a detectable rate, indicating that there are other reactivation pathways present [13].

The [4Fe-4S] electron carrier is highly susceptible to oxidation by superoxide anions/redox cyclers, accounting for the high sensitivity of the *fprB* mutant to these compounds. The sensitivity phenotype could be fully suppressed by the increasing supply of the [Fe-S] clusters through increased expression of genes from the *isc* operon, supporting the possibility that the *fprB* inactivation leads to the decrease in the [4Fe-4S] cluster supply via a decrease in reduced ferredoxin, a possible product of FprB-catalyzed reaction.

### The physiological role of *fprB* in osmotic stress

**Fig 7:**
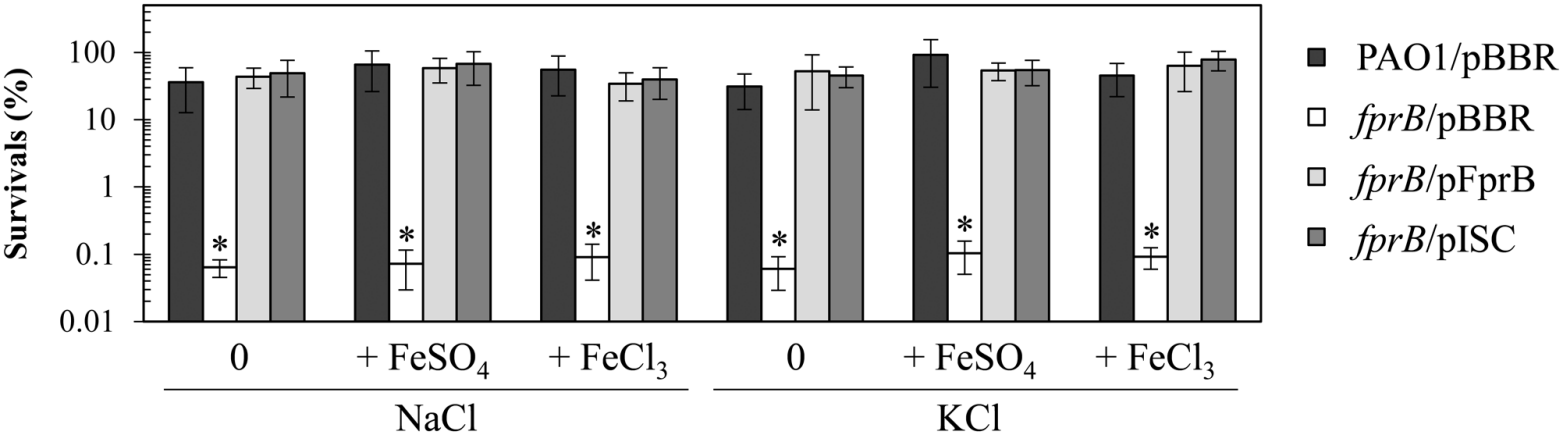
Determination of resistance to high salt conditions. Plate sensitivity assays were performed to determine the levels of resistance of *P*. *aeruginosa* PAO1 and *fprB* mutant harboring empty vector (pBBR), pFprB, or pISC strains under conditions of either high sodium (NaCl, 0.5 M) or high potassium (KCl, 0.5 M) salts with/without ferrous (FeSO_4_, 50 μM) or ferric (FeCl_3_, 50 μM) supplementation. The data shown are the means and SD of percent survival at 18 h incubation from three independent experiments. The asterisk indicates a statistically significant difference (P < 0.01) compared to those of the PAO1/pBBR strain.

The resistance of the *fprB* mutant toward various stress producing substances was determined. *P*. *putida fpr* mutants show growth defects under osmotic stress conditions that are partially complemented by iron supplementation to the growth media, suggesting that iron depletion is responsible for the mutant phenotype [13]. Hence, the role of PAO1 *fprB* in osmotic stress protection was tested. The results of plate sensitivity assays to salt stresses showed that the *fprB* mutant was highly sensitive to NaCl (0.5 M) and KCl (0.5 M) (Fig 7). High salt treatments caused 10^3^-fold (NaCl) and 10^3^-fold (KCl) decreases in percent-survival rates of *fprB* mutants compared with the parent strain PAO1 (Fig 7). The osmotic stress-sensitive phenotype could be fully complemented by expression of functional *fprB* in the *fprB*/pFprB strain (Fig 7). However, supplementing the medium with ferric or ferrous salts in various concentrations (0.05–1 mM) did not complement the osmotic sensitivity of the mutant (Fig 7). The results suggest that in PAO1, salt stress does not lead to intracellular iron depletion. Additional experiments were performed to test whether increased [Fe-S] cluster biogenesis by increased expression of genes from *isc* operon on a plasmid (pISC) could complement the salt sensitivity of the *fprB* mutant. These results showed that *fprB*/pISC expression could fully restore the salt stress hypersensitivity in the *fprB* mutant to the levels attained in PAO1 (Fig 7).

The results support that *fprB* has an important role in PAO1 survival under osmotic stress, similar to the result obtained for *P*. *putida* [13]. Nonetheless, the mechanisms responsible for the phenotype in the closely related bacteria are different, as the osmotic sensitivity could be complemented by increasing the expression of genes from the *isc* operon, indicating that [4Fe-4S] deficiencies in the *P*. *aeruginosa* mutant are likely responsible for the phenotype. In the PAO1 *fprB* mutant, increased sensitivity to high salt stress could result from the decrease in [4Fe-4S] cluster biogenesis and reduction of cluster availability, perhaps by reducing the functionality of [4Fe-4S] cluster-containing proteins that modulate the intracellular Na^+^/K^+^ contents. The reduced functionality of [4Fe-4S] cluster-containing proteins such as RnfB and RnfC, which are known to be involved in the Na^+^-translocation, are likely responsible for the phenotype of *P*. *aeruginosa fprB* mutant [44, 45].

### *fprB* is required for protection against metal toxicity

**Fig 8:**
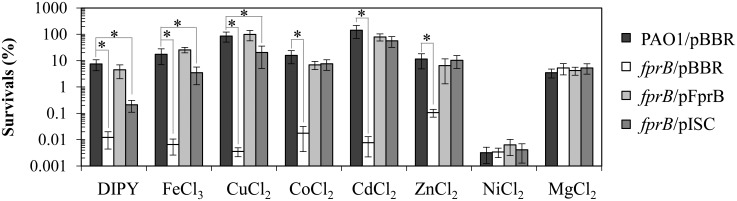
Determination of resistance levels to metals, iron depletion and iron repletion conditions. Plate sensitivity assays were performed to determine the resistance levels of *P*. *aeruginosa* PAO1 and *fprB* mutant harboring empty vector (pBBR), pFprB, or pISC strains against iron-chelating agent 2,2’-dipyridyl (DIPY, 1.2 mM), ferric iron (FeCl_3_, 5.5 mM), copper (CuCl_2_, 4.2 mM), cobalt (CoCl_2_, 0.5 mM), cadmium (CdCl_2_, 0.8 mM), zinc (ZnCl_2_, 5 mM), nickel (NiCl_2_, 2.5 mM), and magnesium (MgCl_2_, 15 mM). The data shown are the means and SD of percent survival at 18 h incubation from three independent experiments. The asterisk indicates a statistically significant difference (P < 0.01) between the two indicated strains.

*P*. *aeruginosa* has the ability to grow in diverse environmental niches. The physiological role of *fprB* in the protection against metal toxicity was also examined. First, the ability of the *fprB* mutant and PAO1 to survive in iron repletion and depletion conditions was examined. Several bacterial Fpr proteins have additional ferric reductase activity, which is important for the iron assimilation process [4, 8]. Exposure to toxic levels of iron (FeCl_3_, 5.5 mM) caused a 10^4^-fold drop in percent survival of the mutant compared to PAO1 (Fig 8). Unexpectedly, growth in iron depletion conditions (induced via inclusion of an intracellular iron chelator, 2,2’-dipyridyl [DIPY], 1.2 mM) also led to a 10^3^-fold drop in the survival rate of the mutant (Fig 8). The ability to survive toxic concentrations of metals was further tested using different metal treatments in PAO1. Treatment of the *fprB* mutant with copper (CuCl_2_, 4.2 mM), cobalt (CoCl_2_, 0.5 mM), cadmium (CdCl_2_, 0.8 mM), and zinc (ZnCl_2_, 5 mM) caused 10^3^- to10^4^-fold reduction in survival percentages compared to the wild type. Treatment of the mutant with nickel (NiCl_2_, 2.5 mM) and magnesium (MgCl_2_, 15 mM), however, did not alter the percent survival compared to wild type. The increased sensitivity of *fprB* mutants to various metals could be fully complemented in the *fprB/*pFprB strain (Fig 8). Clearly, FprB has an important role in protecting cells from metal toxicity, though the question remained whether *fprB* mutant metal sensitivity phenotypes arose from a common defect or a defect in the ability to protect cells against levels of an individual metal. Here, we have shown that both the oxidative and osmotic stress sensitivity of the *fprB* mutant could be partially or wholly alleviated by increased expression of genes from the *isc* operon on a plasmid vector (Figs 6 and 7). Experiments were performed to test whether the expression of genes from the *isc* operon could protect the mutant from metal toxicity. The results of metal treatment on the mutant harboring pISC compared with control strains could be used to group metal treatments into three groups. Expression of genes from the *isc* operon partially complemented iron starvation conditions (from DIPY treatment) to a level 50-fold higher survival than the mutant but still 50-fold lower than that of PAO1 (Fig 8). The *fprB*/pISC showed less than 10-fold lower percent survival than wild type with the second group of metal treatments (repletion of FeCl_3_ and CuCl_2_), even though the mutant itself was highly sensitive (10^3^- to 10^4^-fold) (Fig 8). The mutant sensitivity to the last group of metals, CoCl_2_, CdCl_2_ and ZnCl_2_ treatments, was fully complemented to PAO1 levels in the *fprB*/pISC strain (Fig 8). Expression of pISC in the mutant had no effects on the NiCl_2_ and MgCl_2_ treatments, similar to the *fprB* mutant response to these treatments (Fig 8).

The increased sensitivity of the mutant to high FeCl_3_ treatment and partial complementation by pISC suggests that in the *fprB* mutant, the decreased levels of reduced ferredoxin and the consequent decreased pool of [4Fe-4S] cluster increases iron availability and an insufficient transfer of this excess iron into storage. Therefore, accumulation of labile iron by the addition of excess iron led to cellular toxicity. Bacteria maintain a small pool of labile iron that is strictly controlled to assure sufficient concentration for growth while preventing its toxic accumulation [39, 46]. The [Fe-S] clusters could have an additional role as an iron sink to prevent excess labile iron from creating harmful side effects via the Fenton reaction. This rationale is supported by the observation that the expression of genes from pISC alleviates the sensitivity of the mutant to FeCl_3_ treatment. Expression of genes from pISC increases [Fe-S] cluster assembly, thereby increasing iron sinks that can decrease toxic levels of labile iron pools. Iron depletion is a stressful condition for most organisms due to the requirement of iron in iron-containing cofactors such as heme and [Fe-S] clusters for the activity of many enzymes and proteins [12, 47]. The increased sensitivity of the mutant to iron depletion condition (via DIPY treatment) and partial complementation by *isc* operon expression suggests that low intracellular iron concentration causes reduced [Fe-S] biogenesis and subsequent reduction in functional [Fe-S] cluster-containing proteins. This deficiency could be partially compensated by increasing [Fe-S] biogenesis, although, as expected, increased [Fe-S] biogenesis alone could not fully overcome iron-depleted conditions. In addition, Fpr has a role in iron mobilization from storage [25], which is crucial under iron-depleted conditions.

[Fe-S] clusters and [Fe-S] cluster-containing enzymes/proteins are targets of reactive metals. The metal-[Fe-S] interactions often cause [Fe-S] cluster damage and an associated loss of function in [Fe-S] cluster-containing enzymes/proteins [48]. Copper toxicity has been shown to arise from its reactivity towards labile or solvent-exposed [Fe-S] cluster-containing proteins such as dehydratases, leading to the loss of iron atoms and subsequent loss of enzyme function [49]. Cobalt indirectly inactivates [Fe-S] clusters by reacting with damaged or newly synthesized clusters and forming mixed iron-cobalt-sulfur complexes that cause the loss of protein functions [50]. [Fe-S] cluster-containing enzymes are also targets of cadmium and zinc, and the ability of these metals to react with and damage [Fe-S] clusters correlate with their ability to bind sulfur [51]. The toxicities of these metals were shown to be more severe in the *fprB* mutant that had a [4Fe-4S] cluster biogenesis defect or redox imbalance. Furthermore, the ability of pISC to fully complement the increased metal sensitivity phenotype indicates that decreasing [4Fe-4S] cluster availability was responsible for the mutant increase in metal sensitivity. Treatments of the mutant with either nickel or magnesium ions that are nonreactive to [Fe-S] clusters did not alter the mutant sensitivity levels. This result is consistent with the role of deficient [Fe-S] cluster biogenesis in increasing the sensitivity of the mutant to reactive metals.

## Conclusion

In sum, the regulation and patterns of stress-induced *fprB* expression suggests a correlation between levels of FprB, [4Fe-4S] clusters, and the ability of bacteria to respond to stress. IscR, a global regulator of the [Fe-S] biogenesis, is required for *fprB* expression during these conditions. IscR acts as a transcription activator of *fprB* expression via direct binding to a motif located near the *fprB* gene promoter. Furthermore, IscR provides an important link between cellular [Fe-S] cluster balance and *fprB* expression. Inactivation of *fprB* leads to a reduction in the availability of reduced ferredoxin, which is required for [4Fe-4S] cluster biosynthesis. Phenotypic analysis of the *P*. *aeruginosa fprB* mutant shows increased susceptibility to oxidative, osmotic and metal stress. A common theme among these diverse sources of stress is the ability to induce [Fe-S] cluster damages, leading to either a decrease in the [Fe-S] cluster pool available for [Fe-S] cluster-containing proteins and/or a loss of [Fe-S] clusters from associated proteins and their subsequent inactivation. The inability to mount a proper physiological response to stressful conditions arose from the insufficient pool of [4Fe-4S] clusters for [Fe-S] cluster-containing proteins in the *fprB* mutant, resulting in subsequent malfunction of these proteins and a defective in stress responses. This notion was supported by observations that increasing biogenesis of [Fe-S] clusters by high expression of the *isc* operon alleviated many of the physiological defects of the *fprB* mutant. Overall, the phenotypic analysis highlighted the important roles of [4Fe-4S] clusters in the bacterial stress response.

## Methods

### Bacterial strains, plasmids, and growth conditions

All bacterial strains and plasmids used in this study are listed in Table 1. Both *E*. *coli* and *P*. *aeruginosa* (PAO1) strains were aerobically cultivated in Luria-Bertani (LB) broth at 37°C with shaking at 180 rpm unless otherwise stated. An overnight culture was inoculated into fresh LB medium to give an optical density at 600 nm (OD_600nm_) of 0.1. Exponential phase cells (OD_600nm_ of about 0.6, after 3 h of growth) were used in all experiments.

**Table 1:** Bacterial strains and plasmids used in this study.

Strain or Plasmid	Relevant characteristics	Source or Reference
***P*. *aeruginosa***		
PAO1	Wild type	ATCC15692
*fprB*	*fprB* mutant, derivative of PAO1 in which *fprB* was disrupted by pKNOCK*fprB*, Gm^r^	This study
*Fur*	*fur* mutant	[58]
Δ*iscR*	*iscR* mutant, derivative of PAO1 in which a part of *iscR* was deleted	[32]
*finR*	*finR* mutant, derivative of PAO1 in which *finR* was disrupted by pKNOCK*finR*, Gm^r^	This study
*oxyR*	*oxyR* mutant, derivative of PAO1 in which *oxyR* was disrupted by pKNOCK*oxyR*, Gm^r^	This study
*soxR*	*soxR* mutant, derivative of PAO1 in which *soxR* was disrupted by pKNOCK*soxR*, Gm^r^	[33]
***E*. *coli***		
DH5α	ϕ80d *lac*ZΔM15, *recA*1, *endA*1, *gyrA*96, *thi-*1, *hsdR*17(r_k_^−^, m_k_^+^), *supE*44, *relA*1, *deoR*, Δ(*lacYMA*-*argF*)U169	Stratagene Inc. (USA)
BW20767	*leu*-63::IS*10 recA1 creC510 hsdR17 endA1 zbf*-5 *uidA*(ΔMlu1)::*pir*+ *thi* RP4-2-tet::Mu-1kan::Tn*7*	[59]
**Plasmids**		
pKNOCK_Gm_	Suicide vector, Gm^r^	[53]
pKNOCK*fprB*	pKNOCK_GM_ containing a 247-bp fragment of *fprB* encoding region, Gm^r^	This study
pKNOCK*finR*	pKNOCK_GM_ containing a 284-bp fragment of *finR* encoding region, Gm^r^	This study
pKNOCK*oxyR*	pKNOCK_GM_ containing a 291-bp fragment of *oxyR* encoding region, Gm^r^	This study
pBBR1-MCS4	Medium-copy-number expression vector, Ap^r^	[55]
pFprB	pBBR1MCS-4 containing *fprB*, Ap^r^	This study
pISC	pBBR1MCS-4 containing *iscSUA-hscBA-fdx2-iscX* coding regions, Ap^r^	[32]
pIscR	pBBR1MCS-4 containing *iscR*, Ap^r^	[32]
pUC18-miniTN7T::GM-*lacZ*	Transcriptional fusion vector with Tn7 insertion at a single site on the chromosome	[60]
pP_*fprB*_	pUC18-miniTN7T::GM-*lacZ* with *fprB*-promoter insertion at MCS	This study
pP_*fprB*_SD	pUC18-miniTN7T::GM-*lacZ* with site-directed mutagenic *fprB*-promoter insertion at MCS	This study

Gm^r^, gentamicin resistance; Ap^r^, ampicillin resistance

### Molecular techniques

General molecular techniques including DNA and RNA preparations, DNA cloning, PCR amplification, Southern and Northern analyses and bacterial transformation were performed according to standard protocols [52]. The oligonucleotide primers used in this study are listed in Table 2.

**Table 2:** List of primers used in this study.

Primer	Sequence (5’→3’)	Purpose
BT87	CACTTAACGGCTGACATGG	Reverse primer in pKnock_Gm_
BT543	TGACGCGTCCTCGGTAC	Forward primer in pKnock_Gm_
BT2781	GCCCGCACAAGCGGTGGAG	Forward primer for 16S ribosomal gene
BT2782	ACGTCATCCCCACCTTCCT	Reverse primer for 16S ribosomal gene
BT3046	CCAGCGGGTCGGCATTCC	Forward primer for *soxR* expression
BT3047	AGGCCTGGAGCGACAGGC	Reverse primer for *soxR* expression
BT3334	TAGACGAGGAAGCCTGGATG	Forward primer for *finR* fragment
BT3335	TGTCCCTGGCCAACTGAG	Reverse primer for *finR* fragment
BT3351	ACCCGCCAGCCAGTTGTC	Forward primer for PA2274 expression
BT3352	CACGCTTTTCGCCCCCAG	Reverse primer for PA2274 expression
BT3458	AAGCCCAGCGGCAGCATC	Forward primer for *fprB* fragment
BT3459	GATCGAGACGAACGGCGC	Reverse primer for *fprB* fragment
BT3499	GTGCTTTGCGGGACACTAGG	Forward primer for full-length *fprB*
BT3500	GCTATCCGCCGCTACTGC	Reverse primer for full-length *fprB*
BT3551	GTCAACCGCAAGCGCTGATCG	Forward primer for *fprB* promoter analysis
BT3552	AGTCAGAGGCTGCACGTCGA	Reverse primer for *fprB* promoter analysis
BT3672	GAGCAGATCACCGGCTTC	Forward primer for *anr* expression
BT3673	GCAGTCTTCTTCGACAGCAG	Reverse primer for *anr* expression
BT4156	AGCAGGGCGAATTCGCCG	Forward primer for *narG* expression
BT4157	TCGTGGTTGGGCAGGTCC	Reverse primer for *narG* expression
EBI134	CAGCCGTACATCACCGAGAC	Forward primer for *fprB* promoter analysis
EBI157	GCGGGGATATTTACAAGCCTT	Forward primer for *fprB* promoter analysis with site-directed mutation
EBI158	AAGGCTTGTAAATATCCCCGC	Reverse primer for *fprB* promoter analysis with site-directed mutation
EBI163	TCGGCGCCATCTACACCATC	Forward primer for *oxyR* fragment
EBI164	GCAGGCTCTTGTCGTTGAG	Reverse primer for *oxyR* fragment
M13F	GTAAAACGACGGCCAGT	universal forward primer for expression vector
M13R	AAACAGCTATGACCATG	universal reverse primer for expression vector

### Construction of *P*. *aeruginosa fprB* mutant

Insertional inactivation of *fprB* (PA4615) was performed using the pKNOCK suicide plasmid vector [53]. The internal DNA fragment of the *fprB* coding region was PCR-amplified with BT3458 and BT3459 primers, using PAO1 genomic DNA as template. The 247-bp PCR product was cloned into pKNOCK_Gm_ digested with *SmaI*, generating pKNOCK_Gm_ *fprB*. This recombinant plasmid was introduced into PAO1 by conjugation [54]. The trans-conjugants were selected by the Gm^r^ phenotype. A single homologous recombination event between the *fprB* fragment in the pKNOCK_Gm_ *fprB* and its counterpart on the chromosome causes insertional inactivation of *fprB*. The *fprB* mutant was confirmed by PCR and Southern blot analyses (S2 Fig).

### Construction of *P*. *aeruginosa finR* and *oxyR* mutant

Insertional inactivation of the *finR* (PA3398) and *oxyR* was performed using pKNOCK vector [53]. The DNA fragments of the *finR* and *oxyR* coding regions were PCR- amplified from PAO1genomic DNA with BT3334 and BT3335 primers (for *finR*) and with EBI163 and EBI164 primers (for *oxyR*). The *finR* and *oxyR* mutants were constructed essentially as described for the *fprB* mutant.

### Construction of pFprB

The full-length *fprB* was PCR-amplified from PAO1 genomic DNA with BT3499 and BT3500 primers. The 797-bp PCR product was cloned into the medium-copy-number expression vector pBBR1MCS-4 [55] cut with *SmaI*, yielding pFprB. The insert was sequenced to verify the correctness of the construct.

### Northern blot analysis

RNA samples were prepared from 10 mL of an exponential phase cell cultures treated with/without oxidant for 15 min as previously described [54]. Total RNA isolation, agarose formaldehyde gel electrophoresis, blotting, and hybridization were performed as previously described [52]. A 247-bp fragment of the *fprB* coding region was amplified from pFprB using primers BT3458 and BT3459 and was used as a probe. The labeling of the DNA probe with [α-^32^P]dCTP was performed using a DNA labeling bead (Amersham, GE Healthcare). The results from a representative experiment out of three independent experiments are shown. Densitometric analysis of the blot using ImageScanner III with LabScan 6.0 software (GE Healthcare) was performed to determine fold induction over the uninduced levels.

### End-point RT-PCR

RNA samples were prepared from 10 mL of an exponential phase cell cultures treated with/without oxidant for 15 min as previously described [54]. Total RNA was extracted from oxidant-induced and uninduced cultures. RNA samples were treated with DNase I (Fermentas, Lithuania) according to the manufacturer's protocols. First-strand cDNA synthesis was performed using RevertAid M-MuLV Reverse Transcriptase with random hexamer primers (Fermentas, Lithuania). PCR was performed using 10 ng cDNA and a specific primer pair (BT3458 and BT3459 for *fprB* or BT2781 and BT2782 for the16S rRNA gene, which was used as an internal control) in a *Taq* PCR master mix kit (Fermentas, Lithuania). PCR amplification was carried out with an initial denaturation step at 95°C for 5 min, followed by 28 cycles of denaturation at 95°C for 15 s, annealing at 55°C for 15 s, and extension at 72°C for 15 s, and a final extension step at 72°C for 10 min. RT-PCR products were separated and visualized using agarose (1.8%) gel electrophoresis and ethidium bromide staining. Densitometric analysis of the blot using ImageScanner III with LabScan 6.0 software (GE Healthcare) was performed to determine fold induction over the uninduced levels.

### Real-time RT-PCR

Cell culture conditions, RNA isolation, and reverse transcription was performed as described for end-point RT-PCR. Real-time RT-PCR was conducted using 10 ng cDNA as template, a specific primer pair and KAPA SYBR FAST qPCR kit. The reaction was run on Applied Biosystems StepOnePlus under the following conditions: denaturation at 95°C for 20 s, annealing at 60°C for 30 s, and extension at 60°C for 30 s, for 40 cycles. The specific primer pairs used for *fprB*, *anr*, *narG*, *soxR*, and PA2274 were BT3458-BT3459, BT3672-BT3673, BT4156-BT4157, BT3046-BT3047, and BT3351-BT3352, respectively. The primer pair for the 16S rRNA gene, which was used as the normalizing gene, was BT2781 and BT2782. Relative expression analysis was calculated using StepOne software v2.1 and is expressed as expression-fold change relative to the level of PAO1 wild type grown under uninduced condition. Experiments were repeated independently three times, and the data shown are means and standard deviations (SD).

### Gel mobility shift assay

6×His-tagged IscR from PAO1 was purified as described [33]. Gel mobility shift assays were performed using a labeled probe containing either native or mutagenic *fprB*-promoters amplified from either pP_*fprB*_ or pP_*fprB*_-SD, respectively, as a template and ^32^P-labeled BT3551 and BT3552 primers. Binding reactions were conducted using 3 fmol of labeled probe in 25 μl of reaction buffer containing 20 mM Tris-HCl (pH 8.0), 50 mM KCl, 4 mM MgCl_2_, 0.5 mM EDTA, 0.02 mg ml^−1^ bovine serum albumin (BSA), 5 mM dithiothreitol (DTT), 10% (vol/vol) glycerol, and 200 ng of poly(dI-dC). Various amounts of purified IscR were added, and the reaction mixture was incubated at 25°C for 20 min. Protein-DNA complexes were separated by electrophoresis on a 7% non-denaturing polyacrylamide gel in 0.5x Tris-borate-EDTA buffer at 4°C and were visualized by exposure to X-ray film.

### Enzymatic assays

Crude cell lysates were prepared from 10 mL of an exponential phase cell cultures treated with/without oxidant for 30 min as previously described [54]. Total protein concentration was determined using dye binding method (BioRad, USA). Succinate dehydrogenase activity assay was performed using method previously described [56] and enzyme activity was expressed as ΔA_600_ per min per milligram protein. Aconitase activity assay was carried out using Aconitase Assay Kit (Abcam, UK). One unit of aconitase was defined as the amount of enzyme that isomerizes 1.0 μmol of citrate to isocitrate per min at pH 7.4 at 25°C.

### Plate sensitivity assay

A plate sensitivity assay was performed to determine the oxidant resistance level as previously described [54]. Briefly, exponential phase cells were adjusted to OD_600_ of 0.1 before making 10-fold serial dilutions. Then, 10 μl of each dilution was spotted onto LB agar plate containing appropriate concentrations of testing reagents. The plates were incubated overnight (about 18 h) at 37°C before the colony forming units (CFU) were scored. Percent survival was defined as the CFU on plates containing oxidant divided by the CFU on plates without oxidant and multiply by 100.

### Bacterial killing assay

Killing assay was performed to determine the oxidant killing levels as previously described [54]. Briefly, the exponential phase cultures were adjusted to give an OD_600_ of 0.5 before being treated with appropriate concentrations of oxidants for 30 min and were serially 10-fold diluted. 10 μL of diluted cultures was spot on the LB agar plate and incubated overnight (18 h). Cells survived killing treatments were scored by viable cell count. Percent survival was defined as the number of cells survived treatments divided by the number of cells in untreated control and multiply by 100.

## Supporting Information

S1 Fig Amino acid sequence alignment of Fpr in *P*. *aeruginosa* PAO1. Alignment of *P*. *aeruginosa* FprA and FprB was performed using the CLUSTALW program. Black and light grey boxes indicate the subclass signature amino acid (F for subclass I Fpr and YW for subclass II Fpr) and the FAD-binding domain, respectively. The asterisk, colon, and period symbols indicate identical residues, conserved substitutions, and semi-conserved substitutions, respectively. Number indicates percent identity of the aligned protein with that of FprA.
(TIF)

S2 Fig Southern blot analysis of the *fprB* mutation on *P*. *aeruginosa* genomes in a wild-type PAO1 and a *fprB* mutant. Genomic DNA was extracted and digested with restriction enzyme, *BclI*. Digested DNA was run on 1% agarose gel and transferred into membrane. The membrane was hybridized with radioactive-labelled DNA fragment in the region of *fprB* gene. PAO1 gave a hybridized band of 1560 bp, while the *fprB* mutant gave a 3500-bp band. The radioactive-labelled lambda DNA *EcoRI* + *HindIII* were also presented in the reaction.
(TIF)

## References

[1] Bianchi V, Haggard-Ljungquist E, Pontis E, Reichard P (1995) Interruption of the ferredoxin (flavodoxin) NADP^+^ oxidoreductase gene of *Escherichia coli* does not affect anaerobic growth but increases sensitivity to paraquat. J Bacteriol 177: 4528–4531. 7635836 10.1128/jb.177.15.4528-4531.1995 PMC177208

[2] Medina M, Abagyan R, Gomez-Moreno C, Fernandez-Recio J (2008) Docking analysis of transient complexes: interaction of ferredoxin-NADP^+^ reductase with ferredoxin and flavodoxin. Proteins 72: 848–862. 10.1002/prot.21979 18260112 PMC4162409

[3] Shin M (2004) How is Ferredoxin-NADP reductase involved in the NADP photoreduction of chloroplasts? Photosynth Res 80: 307–313. 16328828 10.1023/B:PRES.0000030456.96329.f9

[4] Yeom J, Jeon CO, Madsen EL, Park W (2009) Ferredoxin-NADP^+^ reductase from *Pseudomonas putida* functions as a ferric reductase. J Bacteriol 191: 1472–1479. 10.1128/JB.01473-08 19114475 PMC2648195

[5] Carrillo N, Ceccarelli EA (2003) Open questions in ferredoxin-NADP^+^ reductase catalytic mechanism. Eur J Biochem 270: 1900–1915. 12709048 10.1046/j.1432-1033.2003.03566.x

[6] Tondo ML, Musumeci MA, Delprato ML, Ceccarelli EA, Orellano EG (2011) Structural-functional characterization and physiological significance of ferredoxin-NADP reductase from *Xanthomonas axonopodis* pv. citri. PLoS One 6: e27124 10.1371/journal.pone.0027124 22096528 PMC3212534

[7] Lee Y, Pena-Llopis S, Kang YS, Shin HD, Demple B, Madsen EL, et al (2006) Expression analysis of the fpr (ferredoxin-NADP^+^ reductase) gene in *Pseudomonas putida* KT2440. Biochem Biophys Res Commun 339: 1246–1254. 16360643 10.1016/j.bbrc.2005.11.135

[8] Takeda K, Sato J, Goto K, Fujita T, Watanabe T, Abo M, et al (2010) *Escherichia coli* ferredoxin-NADP^+^ reductase and oxygen-insensitive nitroreductase are capable of functioning as ferric reductase and of driving the Fenton reaction. Biometals 23: 727–737. 10.1007/s10534-010-9339-8 20407804

[9] Giro M, Carrillo N, Krapp AR (2006) Glucose-6-phosphate dehydrogenase and ferredoxin-NADP(H) reductase contribute to damage repair during the *soxRS* response of *Escherichia coli* . Microbiology 152: 1119–1128. 16549675 10.1099/mic.0.28612-0

[10] Yeom S, Yeom J, Park W (2010) Molecular characterization of FinR, a novel redox-sensing transcriptional regulator in *Pseudomonas putida* KT2440. Microbiology 156: 1487–1496. 10.1099/mic.0.034181-0 20056701

[11] Park W, Pena-Llopis S, Lee Y, Demple B (2006) Regulation of superoxide stress in Pseudomonas putida KT2440 is different from the SoxR paradigm in *Escherichia coli* . Biochem Biophys Res Commun 341: 51–56. 16412384 10.1016/j.bbrc.2005.12.142

[12] Yeom J, Imlay JA, Park W (2010) Iron homeostasis affects antibiotic-mediated cell death in *Pseudomonas* species. J Biol Chem 285: 22689–22695. 10.1074/jbc.M110.127456 20479007 PMC2903419

[13] Lee Y, Yeom J, Kang YS, Kim J, Sung JS, Jeon CO, et al (2007) Molecular characterization of *fprB* (ferredoxin-NADP^+^ reductase) in *Pseudomonas putida* KT2440. J Microbiol Biotechnol 17: 1504–1512. 18062229

[14] Yan R, Adinolfi S, Pastore A (2015) Ferredoxin, in conjunction with NADPH and ferredoxin-NADP reductase, transfers electrons to the IscS/IscU complex to promote iron-sulfur cluster assembly. Biochim Biophys Acta. 10.1016/j.bbapap.2015.02.002 PMC4547094 25688831

[15] Robbins AH, Stout CD (1989) Structure of activated aconitase: formation of the [4Fe-4S] cluster in the crystal. Proc Natl Acad Sci USA 86: 3639–43. 2726740 10.1073/pnas.86.10.3639 PMC287193

[16] Johnson MK, Morningstar JE, Bennett DE, Ackrell BA, Kearney EB (1985) Magnetic circular dichroism studies of succinate dehydrogenase: Evidence for [2Fe-2S], [3Fe-xS], and [4Fe-4S] centers in reconstitutively active enzyme. J Biol Chem 260: 7368–7378. 2987254

[17] Khoroshilova N, Popescu C, Munck E, Beinert H, Kiley PJ (1997) Iron-sulfur cluster disassembly in the FNR protein of *Escherichia coli* by O_2_: [4Fe-4S] to [2Fe-2S] conversion with loss of biological activity. Proc Natl Acad Sci USA 94: 6087–6092. 9177174 10.1073/pnas.94.12.6087 PMC21006

[18] Arai H, Kodama T, Igarashi Y (1997) Cascade regulation of the two CRP/FNR-related transcriptional regulators (ANR and DNR) and the denitrification enzymes in *Pseudomonas aeruginosa* . Mol Microbiol 25: 1141–1148. 9350869 10.1046/j.1365-2958.1997.5431906.x

[19] Schreiber K, Krieger R, Benkert B, Eschbach M, Arai H, Schobert M, et al (2007) The anaerobic regulatory network required for *Pseudomonas aeruginosa* nitrate respiration. J Bacteriol 189: 4310–4314. 17400734 10.1128/JB.00240-07 PMC1913380

[20] Yao H, Jepkorir G, Lovell S, Nama PV, Weeratunga S, Battaile KP, et al (2011) Two distinct ferritin-like molecules in *Pseudomonas aeruginosa*: the product of the *bfrA* gene is a bacterial ferritin (FtnA) and not a bacterioferritin (Bfr). Biochemistry 50: 5236–5248. 10.1021/bi2004119 21574546 PMC3130351

[21] Wang A, Zeng Y, Han H, Weeratunga S, Morgan BN, Moënne-Loccoz P, et al (2007) Biochemical and structural characterization of *Pseudomonas aeruginosa* Bfd and FPR: ferredoxin NADP^+^ reductase and not ferredoxin is the redox partner of heme oxygenase under iron-starvation conditions. Biochemistry 46: 12198–12211. 17915950 10.1021/bi7013135

[22] Stover CK, Pham XQ, Erwin AL, Mizoguchi SD, Warrener P, Hickey MJ, et al (2000) Complete genome sequence of *Pseudomonas aeruginosa* PAO1, an opportunistic pathogen. Nature 406: 959–964. 10984043 10.1038/35023079

[23] Sridhar Prasad G, Kresge N, Muhlberg AB, Shaw A, Jung YS, Burgess BK, et al (1998) The crystal structure of NADPH:ferredoxin reductase from *Azotobacter vinelandii* . Protein Sci 7: 2541–2549. 9865948 10.1002/pro.5560071207 PMC2143901

[24] Krapp AR, Rodriguez RE, Poli HO, Paladini DH, Palatnik JF, Carrillo N (2002) The flavoenzyme ferredoxin (flavodoxin)-NADP(H) reductase modulates NADP(H) homeostasis during the *soxRS* response of *Escherichia coli* . J Bacteriol 184: 1474–1480. 11844783 10.1128/JB.184.5.1474-1480.2002 PMC134851

[25] Weeratunga SK, Gee CE, Lovell S, Zeng Y, Woodin CL, Rivera M (2009) Binding of *Pseudomonas aeruginosa* apobacterioferritin-associated ferredoxin to bacterioferritin B promotes heme mediation of electron delivery and mobilization of core mineral iron. Biochemistry 48: 7420–7431. 10.1021/bi900561a 19575528 PMC2737084

[26] Palma M, Zurita J, Ferreras JA, Worgall S, Larone DH, Shi L, et al (2005) *Pseudomonas aeruginosa* SoxR does not conform to the archetypal paradigm for SoxR-dependent regulation of the bacterial oxidative stress adaptive response. Infect Immun 73: 2958–2966. 15845502 10.1128/IAI.73.5.2958-2966.2005 PMC1087365

[27] Eiamphungporn W, Charoenlap N, Vattanaviboon P, Mongkolsuk S (2006) *Agrobacterium tumefaciens soxR* is involved in superoxide stress protection and also directly regulates superoxide-inducible expression of itself and a target gene. J Bacteriol 188: 8669–8673. 17041041 10.1128/JB.00856-06 PMC1698218

[28] Mahavihakanont A, Charoenlap N, Namchaiw P, Eiamphungporn W, Chattrakarn S, Vattanaviboon P, et al (2012) Novel roles of SoxR, a transcriptional regulator from *Xanthomonas campestris*, in sensing redox-cycling drugs and regulating a protective gene that have overall implications for bacterial stress physiology and virulence on a host plant. J Bacteriol 194: 209–217. 10.1128/JB.05603-11 22056938 PMC3256661

[29] Kobayashi K, Tagawa S (2004) Activation of SoxR-dependent transcription in *Pseudomonas aeruginosa* . J Biochem 136: 607–615. 15632300 10.1093/jb/mvh168

[30] Ochsner UA, Vasil ML, Alsabbagh E, Parvatiyar K, Hassett DJ (2000) Role of the *Pseudomonas aeruginosa oxyR-recG* operon in oxidative stress defense and DNA repair: OxyR-dependent regulation of *katB-ankB*, *ahpB*, and *ahpC-ahpF* . J Bacteriol 182: 4533–4544. 10913087 10.1128/jb.182.16.4533-4544.2000 PMC94625

[31] Lewis TA, Glassing A, Harper J, Franklin MJ (2013) Role for ferredoxin:NAD(P)H oxidoreductase (FprA) in sulfate assimilation and siderophore biosynthesis in *Pseudomonads* . J Bacteriol 195: 3876–3887. 10.1128/JB.00528-13 23794620 PMC3754593

[32] Romsang A, Duang-Nkern J, Leesukon P, Saninjuk K, Vattanaviboon P, Mongkolsuk S (2014) The iron-sulphur cluster biosynthesis regulator IscR contributes to iron homeostasis and resistance to oxidants in *Pseudomonas aeruginosa* . PLoS One 9: e86763 10.1371/journal.pone.0086763 24466226 PMC3899308

[33] Somprasong N, Jittawuttipoka T, Duang-Nkern J, Romsang A, Chaiyen P, Schweizer HP, et al (2012) *Pseudomonas aeruginosa* thiol peroxidase protects against hydrogen peroxide toxicity and displays atypical patterns of gene regulation. J Bacteriol 194: 3904–3912. 10.1128/JB.00347-12 22609922 PMC3416540

[34] Giel JL, Rodionov D, Liu M, Blattner FR, Kiley PJ (2006) IscR-dependent gene expression links iron-sulphur cluster assembly to the control of O_2_-regulated genes in *Escherichia coli* . Mol Microbiol 60: 1058–1075. 16677314 10.1111/j.1365-2958.2006.05160.x

[35] Rajagopalan S, Teter SJ, Zwart PH, Brennan RG, Phillips KJ, Kiley PJ (2013) Studies of IscR reveal a unique mechanism for metal-dependent regulation of DNA binding specificity. Nat Struct Mol Biol 20: 740–747. 10.1038/nsmb.2568 23644595 PMC3676455

[36] Nesbit AD, Giel JL, Rose JC, Kiley PJ (2009) Sequence-specific binding to a subset of IscR-regulated promoters does not require IscR Fe-S cluster ligation. J Mol Biol 387: 28–41. 10.1016/j.jmb.2009.01.055 19361432 PMC2709974

[37] Membrillo-Hernandez J, Coopamah MD, Anjum MF, Stevanin TM, Kelly A, Hughes MN, et al (1999) The flavohemoglobin of *Escherichia coli* confers resistance to a nitrosating agent, a "Nitric oxide Releaser," and paraquat and is essential for transcriptional responses to oxidative stress. J Biol Chem 274: 748–754. 9873011 10.1074/jbc.274.2.748

[38] Loprasert S, Sallabhan R, Whangsuk W, Mongkolsuk S (2003) Compensatory increase in *ahpC* gene expression and its role in protecting *Burkholderia pseudomallei* against reactive nitrogen intermediates. Arch Microbiol 180: 498–502. 14614594 10.1007/s00203-003-0621-9

[39] Ayala-Castro C, Saini A, Outten FW (2008) Fe-S cluster assembly pathways in bacteria. Microbiol Mol Biol Rev 72: 110–125, table of contents. 10.1128/MMBR.00034-07 18322036 PMC2268281

[40] Py B, Barras F (2010) Building Fe-S proteins: bacterial strategies. Nat Rev Microbiol 8: 436–446. 10.1038/nrmicro2356 20467446

[41] Jang S, Imlay JA (2010) Hydrogen peroxide inactivates the *Escherichia coli* Isc iron-sulphur assembly system, and OxyR induces the Suf system to compensate. Mol Microbiol 78: 1448–1467. 10.1111/j.1365-2958.2010.07418.x 21143317 PMC3051806

[42] Daung-nkern J, Vattanaviboon P, Mongkolsuk S (2010) Inactivation of *nfuA* enhances susceptibility of *Pseudomonas aeruginosa* to fluoroquinolone antibiotics. J Antimicrob Chemother 65: 1831–1832. 10.1093/jac/dkq194 20525732

[43] Romsang A, Leesukon P, Duangnkern J, Vattanaviboon P, Mongkolsuk S (2015) Mutation of the gene encoding monothiol glutaredoxin (GrxD) in *Pseudomonas aeruginosa* increases its susceptibility to polymyxins. Int J Antimicrob Agents 45: 314–318. 10.1016/j.ijantimicag.2014.10.024 25593012

[44] Jouanneau Y, Jeong HS, Hugo N, Meyer C, Willison JC (1998) Overexpression in *Escherichia coli* of the *rnf* genes from *Rhodobacter capsulatus*: characterization of two membrane-bound iron-sulfur proteins. Eur J Biochem 251: 54–64. 9492268 10.1046/j.1432-1327.1998.2510054.x

[45] Biegel E, Muller V (2010) Bacterial Na^+^-translocating ferredoxin:NAD^+^ oxidoreductase. Proc Natl Acad Sci USA 107: 18138–18142. 10.1073/pnas.1010318107 20921383 PMC2964206

[46] Thorgersen MP, Downs DM (2009) Oxidative stress and disruption of labile iron generate specific auxotrophic requirements in *Salmonella enterica* . Microbiology 155: 295–304. 10.1099/mic.0.020727-0 19118370 PMC6756756

[47] Cornelis P, Wei Q, Andrews SC, Vinckx T (2011) Iron homeostasis and management of oxidative stress response in bacteria. Metallomics 3: 540–549. 10.1039/c1mt00022e 21566833

[48] Roche B, Aussel L, Ezraty B, Mandin P, Py B, Barras F. (2013) Iron/sulfur proteins biogenesis in prokaryotes: formation, regulation and diversity. Biochim Biophys Acta 1827: 455–469. 10.1016/j.bbabio.2012.12.010 23298813

[49] Macomber L, Imlay JA (2009) The iron-sulfur clusters of dehydratases are primary intracellular targets of copper toxicity. Proc Natl Acad Sci USA 106: 8344–8349. 10.1073/pnas.0812808106 19416816 PMC2688863

[50] Ranquet C, Ollagnier-de-Choudens S, Loiseau L, Barras F, Fontecave M (2007) Cobalt stress in *Escherichia coli*: the effect on the iron-sulfur proteins. J Biol Chem 282: 30442–30451. 17642475 10.1074/jbc.M702519200

[51] Xu FF, Imlay JA (2012) Silver(I), mercury(II), cadmium(II), and zinc(II) target exposed enzymic iron-sulfur clusters when they toxify *Escherichia coli* . Appl Environ Microbiol 78: 3614–3621. 10.1128/AEM.07368-11 22344668 PMC3346352

[52] Sambrook J, Russell DW (2001) Molecular cloning: a laboratory manual. 3rd ed Cold Spring Harbor Laboratory Press, Cold Spring Harbor, NY.

[53] Alexeyev MF (1999) The pKNOCK series of broad-host-range mobilizable suicide vectors for gene knockout and targeted DNA insertion into the chromosome of gram-negative bacteria. Biotechniques 26: 824–826, 828 10337469 10.2144/99265bm05

[54] Romsang A, Atichartpongkul S, Trinachartvanit W, Vattanaviboon P, Mongkolsuk S (2013) Gene expression and physiological role of *Pseudomonas aeruginosa* methionine sulfoxide reductases during oxidative stress. J Bacteriol 195: 3299–3308. 10.1128/JB.00167-13 23687271 PMC3719549

[55] Kovach ME, Phillips RW, Elzer PH, Roop RM 2nd, Peterson KM (1994) pBBR1MCS: a broad-host-range cloning vector. Biotechniques 16: 800–802. 8068328

[56] Enos-Berlage JL, Downs DM (1997) Mutations in sdh (succinate dehydrogenase genes) alter the thiamine requirement of *Salmonella typhimurium* . J Bacteriol 179: 3989–3996. 9190816 10.1128/jb.179.12.3989-3996.1997 PMC179209

[57] Larkin MA, Blackshields G, Brown NP, Chenna R, McGettigan PA, McWilliam H, et al (2007) Clustal W and Clustal X version 2.0. Bioinformatics 23: 2947–2948. 17846036 10.1093/bioinformatics/btm404

[58] Hassett DJ, Sokol PA, Howell ML, Ma JF, Schweizer HT, Ochsner U, et al (1996) Ferric uptake regulator (Fur) mutants of *Pseudomonas aeruginosa* demonstrate defective siderophore-mediated iron uptake, altered aerobic growth, and decreased superoxide dismutase and catalase activities. J Bacteriol 178: 3996–4003. 8763923 10.1128/jb.178.14.3996-4003.1996 PMC178152

[59] Metcalf WW, Jiang W, Daniels LL, Kim SK, Haldimann A, Wanner BL (1996) Conditionally replicative and conjugative plasmids carrying *lacZ* alpha for cloning, mutagenesis, and allele replacement in bacteria. Plasmid 35: 1–13. 8693022 10.1006/plas.1996.0001

[60] Choi KH, Schweizer HP (2006) mini-Tn7 insertion in bacteria with single attTn7 sites: example *Pseudomonas aeruginosa* . Nat Protoc 1: 153–161. 17406227 10.1038/nprot.2006.24

