# Supplementary figures and images for: Pseudomonas aeruginosa IscR-Regulated Ferredoxin NADP(+) Reductase Gene (fprB) Functions in Iron-Sulfur Cluster Biogenesis and Multiple Stress Response
Source: PLoS One. 2015 Jul 31;10(7):e0134374. doi: 10.1371/journal.pone.0134374 (PMC4521836; doi:10.1371/journal.pone.0134374)

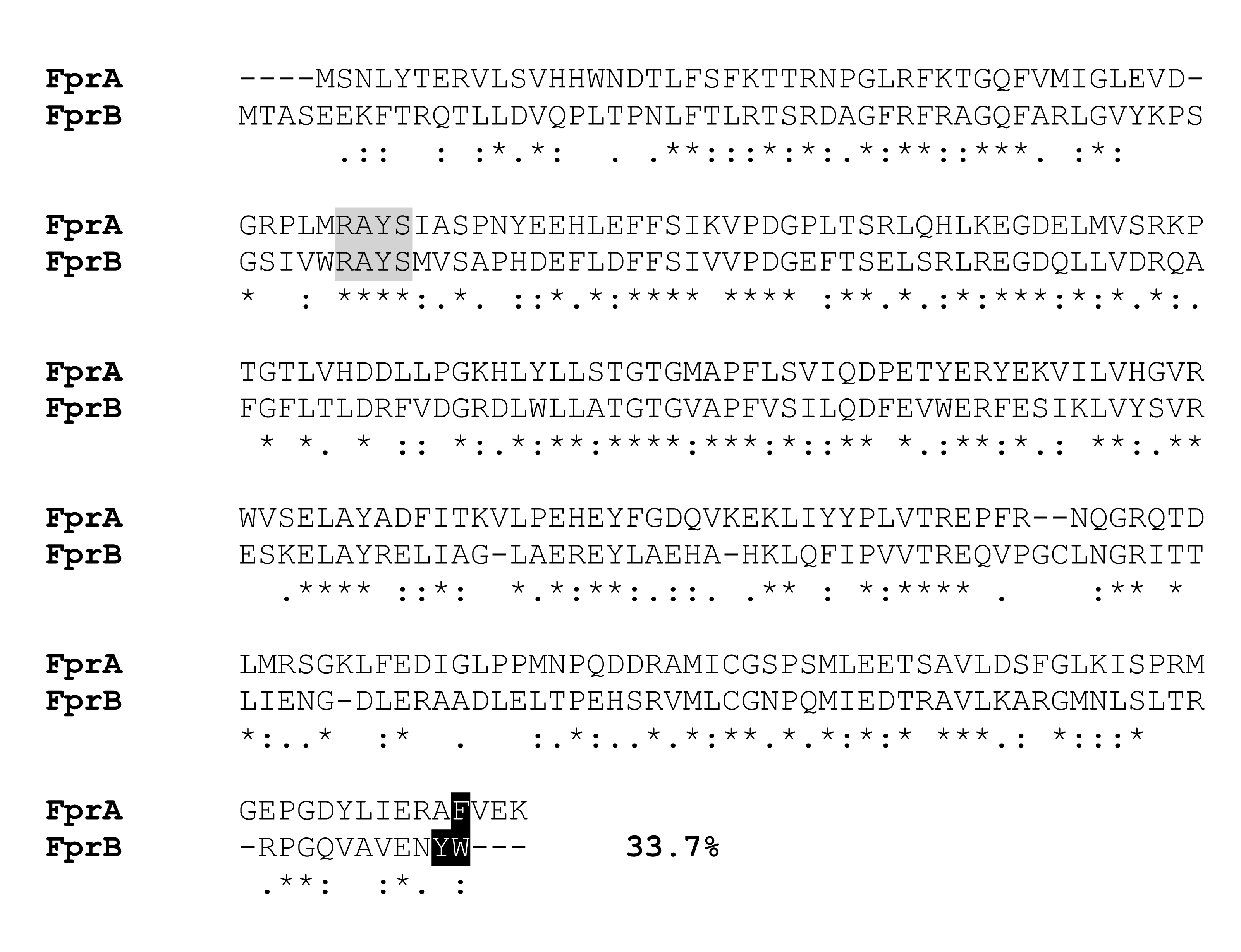

Supplement: S1 Fig — Alignment of P. aeruginosa FprA and FprB was performed using the CLUSTALW program. Black and light grey boxes indicate the subclass signature amino acid (F for subclass I Fpr and YW for subclass II Fpr) and the FAD-binding domain, respectively. The asterisk, colon, and period symbols indicate identical residues, conserved substitutions, and semi-conserved substitutions, respectively. Number indicates percent identity of the aligned protein with that of FprA. (TIF) [file pone.0134374.s001.tif]

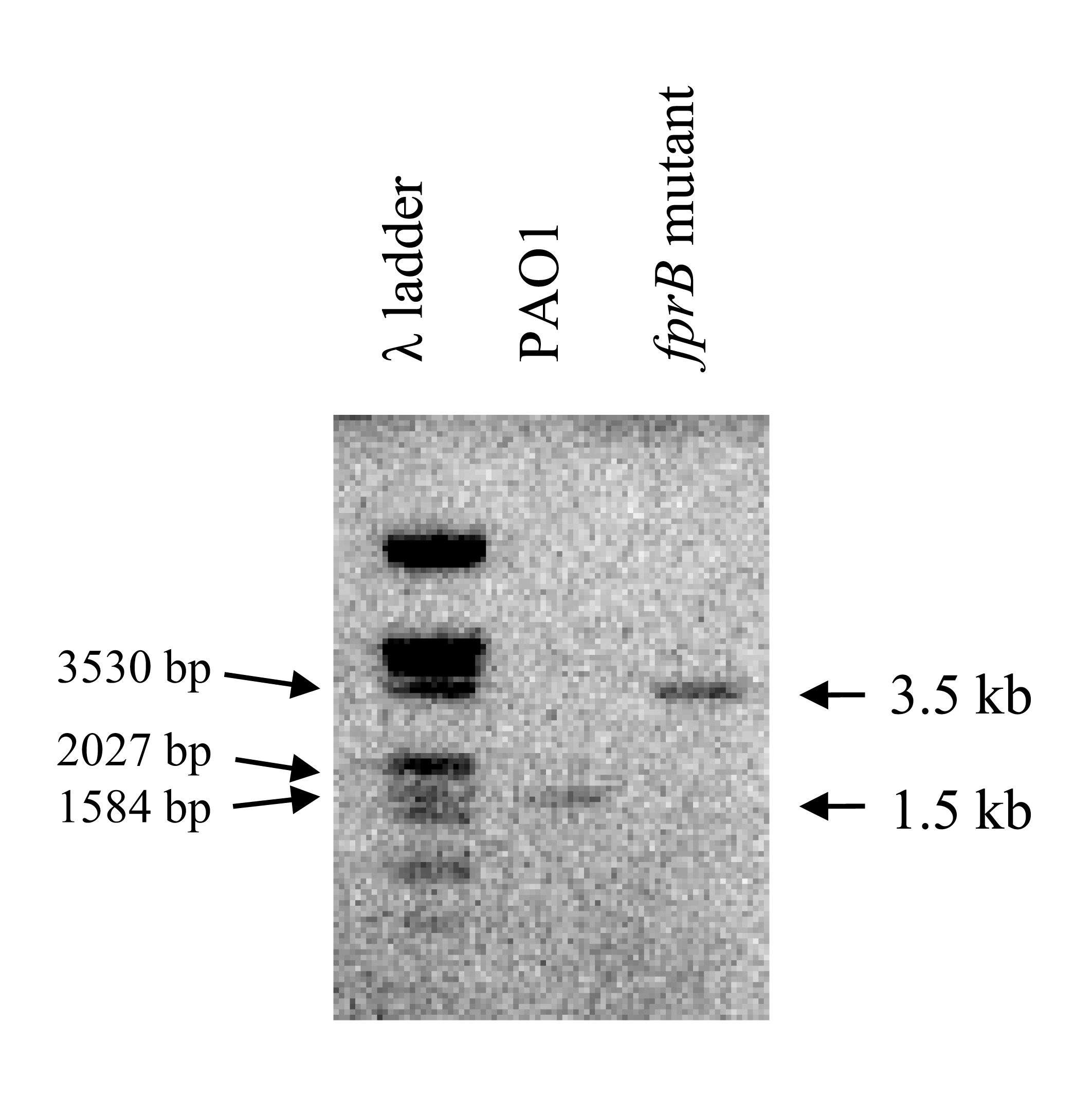

Supplement: S2 Fig — Genomic DNA was extracted and digested with restriction enzyme, BclI. Digested DNA was run on 1% agarose gel and transferred into membrane. The membrane was hybridized with radioactive-labelled DNA fragment in the region of fprB gene. PAO1 gave a hybridized band of 1560 bp, while the fprB mutant gave a 3500-bp band. The radioactive-labelled lambda DNA EcoRI + HindIII were also presented in the reaction. (TIF) [file pone.0134374.s002.tif]
